# Could a Mediterranean Diet Modulate Alzheimer’s Disease Progression? The Role of Gut Microbiota and Metabolite Signatures in Neurodegeneration

**DOI:** 10.3390/foods14091559

**Published:** 2025-04-29

**Authors:** Alice N. Mafe, Dietrich Büsselberg

**Affiliations:** 1 Department of Biological Sciences, Faculty of Sciences, Taraba State University, Main Campus, Jalingo 660101, Taraba State, Nigeria; mafealice1991@gmail.com; 2 Weill Cornell Medicine-Qatar, Education City, Qatar Foundation, Doha Metropolitan Area, Ar-Rayyan P.O. Box 22104, Qatar

**Keywords:** cognitive decline, neuroinflammation, microbial metabolites, brain aging, dietary interventions

## Abstract

Neurodegenerative disorders such as Alzheimer’s disease (AD), the most common form of dementia, represent a growing global health crisis, yet current treatment strategies remain primarily palliative. Recent studies have shown that neurodegeneration through complex interactions within the gut–brain axis largely depends on the gut microbiota and its metabolites. This review explores the intricate molecular mechanisms linking gut microbiota dysbiosis to cognitive decline, emphasizing the impact of microbial metabolites, including short-chain fatty acids (SCFAs), bile acids, and tryptophan metabolites, on neuroinflammation, blood–brain barrier (BBB) integrity, and amyloid-β and tau pathology. The paper highlights major microbiome signatures associated with Alzheimer’s disease, detailing their metabolic pathways and inflammatory crosstalk. Dietary interventions have shown promise in modulating gut microbiota composition, potentially mitigating neurodegenerative processes. This review critically examines the influence of dietary patterns, such as the Mediterranean and Western diets, on microbiota-mediated neuroprotection. Bioactive compounds like prebiotics, omega-3 fatty acids, and polyphenols exhibit neuroprotective effects by modulating gut microbiota and reducing neuroinflammation. Furthermore, it discusses emerging microbiome-based therapeutic strategies, including probiotics, prebiotics, postbiotics, and fecal microbiota transplantation (FMT), as potential interventions for slowing Alzheimer’s progression. Despite these advances, several knowledge gaps remain, including interindividual variability in microbiome responses to dietary interventions and the need for large-scale, longitudinal studies. The study proposes an integrative, precision medicine approach, incorporating microbiome science into Alzheimer’s treatment paradigms. Ultimately, cognizance of the gut–brain axis at a mechanistic level could unlock novel therapeutic avenues, offering a non-invasive, diet-based strategy for managing neurodegeneration and improving cognitive health.

## 1. Introduction

Neurodegenerative diseases, particularly Alzheimer’s disease, represent a growing global health crisis. With an aging population, the incidence of these conditions is steadily rising, placing an enormous burden on healthcare systems and caregivers [1]. According to the World Health Organization, more than 55 million people live with dementia worldwide, with Alzheimer’s disease accounting for 60–70% of these cases. This number is projected to nearly triple, reaching 139 million by 2050, due to increasing life expectancy and demographic shifts [2]. The global economic cost of dementia was estimated at over USD 1.3 trillion in 2023, a figure expected to surge to USD 2.8 trillion by 2030, further straining healthcare resources, especially in low- and middle-income countries where cases are rising most rapidly [3]. These alarming statistics underscore the urgent need for innovative strategies that can prevent, delay, or mitigate the progression of Alzheimer’s and related dementias. The economic cost of managing Alzheimer’s disease alone runs into hundreds of billions of dollars annually, yet there remains no cure [4]. Current treatment strategies focus mainly on symptom management, often providing limited benefits in slowing disease progression [5]. This growing hurdle highlights the urgent need for innovative approaches beyond conventional pharmaceutical interventions [6]. One emerging area of interest is the role of the gut–brain axis in neurodegeneration. The gut microbiota, a complex ecosystem of trillions of microorganisms residing in the intestine, is critical in maintaining overall health, including brain function [7]. Research has shown that disruptions in the gut microbiome, often called dysbiosis, can contribute to systemic inflammation, metabolic imbalances, and ultimately, cognitive decline [8]. Evidence suggests that an altered gut microbiome in individuals with Alzheimer’s can influence disease onset and progression through microbial metabolites, immune signaling, and gut-derived neurotoxins [9].

Despite these intriguing connections, several fundamental questions remain unanswered. Which specific gut microbiota-derived metabolites contribute to neurodegeneration? Can dietary interventions reshape the microbiome in a way that slows cognitive decline? Tackling these questions could open new doors for microbiome-based strategies to prevent or mitigate Alzheimer’s disease. At the molecular level, gut microbiota-derived metabolites, such as SCFAs and bile acids, have been implicated in either neuroprotection or neurotoxicity. SCFAs, especially butyrate, are essential for reducing neuroinflammation and maintaining blood–brain barrier integrity [10]. Conversely, an imbalance in bile acid metabolism has been linked to neuronal dysfunction and increased amyloid-beta accumulation [11]. Equally, gut microbes influence neuroinflammatory pathways by modulating cytokines like TNF-α, IL-1, and IL-6, which play a significant role in Alzheimer’s pathology [12]. These mechanisms are essential in determining whether dietary modifications can harness beneficial gut metabolites to counteract neurodegeneration. This review hypothesizes that dietary modulation, particularly through Mediterranean dietary patterns, can influence gut microbiota composition and metabolite production in ways that mitigate neurodegenerative processes characteristic of Alzheimer’s disease.

## 2. Microbiome Signatures in Alzheimer’s Disease

### 2.1. Gut Microbiota Dysbiosis in Alzheimer’s Disease

**Figure 1:**
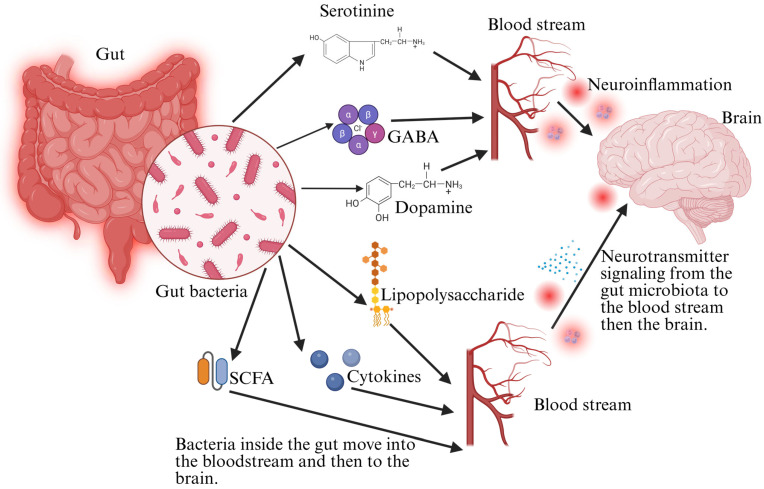
Gut–brain axis and neurodegeneration (created with BioRender. Busselberg, D. (2025) https://app.biorender.com/illustrations/67ec0b168953a880ac2b9ed4).

An essential component of maintaining general health, including brain function, is the gut flora, through its interactions with the immune, metabolic, and nervous systems [13]. However, in individuals with Alzheimer’s disease and dementia, significant alterations in gut microbial composition, referred to as dysbiosis, have been observed [14]. These changes often involve reduced beneficial bacteria and increased pathogenic or pro-inflammatory microbial species, which can contribute to neurodegenerative processes [15]. Research has indicated that those who have Alzheimer’s typically possess lower microbial diversity than healthy individuals. A balanced microbiome, rich in various bacterial species, is essential for producing beneficial metabolites like SCFAs, which help regulate neuroinflammation and maintain the integrity of the blood–brain obstruction [16]. However, in Alzheimer’s patients, a decrease in SCFA-producing bacteria, such as *Faecalibacterium* sp., *Bifidobacterium* sp., and *Akkermansia* sp., has been reported. This reduction can lead to increased neuronal injury, inflammation, and oxidative stress, all contributing to neurocognitive dysfunction [17]. Also, specific bacterial taxa are more abundant in individuals with neurodegenerative diseases. For example, increased levels of Proteobacteria and Bacteroides have been linked to higher production of lipopolysaccharides (LPS), a bacterial endotoxin known to trigger systemic inflammation and disrupt blood–brain barrier function [18]. The presence of these harmful microbial metabolites in circulation can promote neuroinflammation, exacerbate amyloid-beta accumulation, and accelerate the progression of dementia. The observed differences in gut microbiota between healthy individuals and those with Alzheimer’s highlight the potential role of the gut–brain axis in disease pathology [19]. These microbial alterations might offer information about new diagnostic markers and therapeutic targets for neurodegenerative conditions. Further research is needed to determine whether dietary interventions, probiotics, or microbiome-based therapies could restore microbial balance and potentially slow the progression of Alzheimer’s disease. The gut–brain axis has been linked to neurodegeneration through various microbial and metabolic pathways (Figure 1).

### 2.2. Gut Microbiota-Derived Metabolites and Neurodegeneration: Molecular Mechanisms of SCFAs, Tryptophan, and Bile Acid Pathways

The gut microbiota profoundly influences neurodegenerative processes by producing bioactive metabolites that modulate the central nervous system (CNS) function. These metabolites can cross the blood–brain barrier (BBB) or signal through neuroimmune and neuroendocrine pathways [20]. Among the most extensively studied are short-chain fatty acids (SCFAs), tryptophan-derived metabolites, and bile acids, which have been implicated in the pathophysiology of Alzheimer’s disease (AD) [21]. Dysregulation of these metabolic pathways contributes to neuroinflammation, BBB disruption, oxidative stress, and the accumulation of neurotoxic proteins.

#### 2.2.1. SCFAs: Modulation of Neuroinflammation, BBB Integrity, and Amyloid-β Clearance

SCFAs are principally acetate, propionate, and butyrate, fermentation products of dietary fibers by gut microbiota. Butyrate, in particular, exhibits potent neuroprotective properties [22]. It is predominantly produced by beneficial bacterial taxa such as *Faecalibacterium* sp., *Roseburia* sp., and *Akkermansia* sp. [23]. As a histone deacetylase (HDAC) inhibitor, butyrate modulates gene expression to suppress pro-inflammatory pathways (e.g., NF-κB, IL-6), enhance mitochondrial function, and support synaptic plasticity [24]. One of butyrate’s key roles is maintaining BBB integrity by upregulating tight junction proteins (e.g., claudins, occludins), thereby limiting permeability and preventing the influx of neurotoxic agents [25]. In contrast, dysbiosis-related increases in microbial endotoxins, such as lipopolysaccharide (LPS), activate toll-like receptor 4 (TLR4) on endothelial cells, disrupt tight junctions, and promote neuroinflammatory cascades [26]. Butyrate facilitates amyloid-β (Aβ) clearance by enhancing microglial phagocytic activity. This effect is mediated through HDAC inhibition, which stimulates gene expression in immune regulation and resolution of inflammation [27]. In AD patients, diminished butyrate-producing bacteria correlate with reduced SCFA concentrations, elevated systemic LPS, and impaired Aβ clearance, underscoring the therapeutic relevance of restoring SCFA homeostasis [28].

#### 2.2.2. Tryptophan Metabolism: A Balance Between Neuroprotection and Neurotoxicity

Gut microbiota tightly regulate tryptophan metabolism, influencing serotonergic pathways and immune function. Under physiological conditions, microbial activity directs tryptophan toward the synthesis of serotonin and indole derivatives, which are neuroprotective and anti-inflammatory [29]. However, in AD, metabolic flux shifts toward the kynurenine pathway, favoring the production of neurotoxic metabolites such as quinolinic acid, which is a potent NMDA receptor agonist that induces excitotoxicity and neuronal injury [30]. Microbial dysbiosis exacerbates this shift, enhancing the generation of pro-inflammatory kynurenine derivatives while diminishing beneficial indole compounds. This imbalance contributes to microglial activation and chronic neuroinflammation. Interventions that modulate microbial composition or dietary intake to rebalance tryptophan metabolism may offer neuroprotective benefits [31].

The serotonergic branch of tryptophan metabolism facilitates the synthesis of serotonin (5-hydroxytryptamine), a key neurotransmitter that governs mood regulation, cognitive function, and neuroplasticity. Within the gut–brain axis, serotonin also plays essential roles in gastrointestinal motility and immune balance [32]. The gut microbiota actively influences this pathway by stimulating tryptophan hydroxylase activity and promoting peripheral serotonin production [33]. In a healthy physiological state, optimal serotonin levels help to dampen inflammation, inhibit microglial activation, and shield neurons from damage. However, in neurodegenerative diseases such as Alzheimer’s, a metabolic shift away from the serotonergic route weakens these protective effects, potentially accelerating cognitive impairment and emotional dysregulation [34]. In contrast, the neurotoxic arm of the kynurenine pathway involves the breakdown of tryptophan into metabolites like 3-hydroxykynurenine and quinolinic acid. Quinolinic acid, a potent NMDA receptor agonist, induces excitotoxicity by triggering excessive calcium influx, oxidative stress, and eventual neuronal cell death [35]. Inflammatory states, particularly those seen in Alzheimer’s disease, which upregulate the enzyme indoleamine 2,3-dioxygenase (IDO), predominantly in activated microglia and astrocytes, thereby diverting tryptophan metabolism toward this deleterious pathway [36]. The accumulation of quinolinic acid not only exerts direct neurotoxic effects but also amplifies neuroinflammation by stimulating glial activation and pro-inflammatory cytokine release, perpetuating a cycle of progressive neural damage

#### 2.2.3. Bile Acids and the Gut–Liver–Brain Axis

Beyond their classical role in lipid metabolism, bile acids function as neuromodulatory molecules within the gut–liver–brain axis. Their synthesis and conversion into secondary bile acids are microbiota-dependent [37]. In AD, altered bile acid profiles, particularly elevated levels of cytotoxic derivatives, are associated with increased oxidative stress, neuroinflammation, and impaired neuronal signaling. Bile acid receptors such as farnesoid X receptor (FXR) and Takeda G protein-coupled receptor 5 (TGR5) mediate critical neurophysiological processes, including inflammation regulation, mitochondrial dynamics, and synaptic function [38]. Dysbiosis-induced disturbances in bile acid metabolism can impair these receptor-mediated signaling pathways, thereby contributing to neurodegenerative progression [39]. Modulating gut microbiota to restore balanced bile acid profiles represents a promising therapeutic avenue for cognitive preservation.

### 2.3. Molecular Crosstalk Between Gut Microbes and Neuroinflammatory Pathways

**Figure 2:**
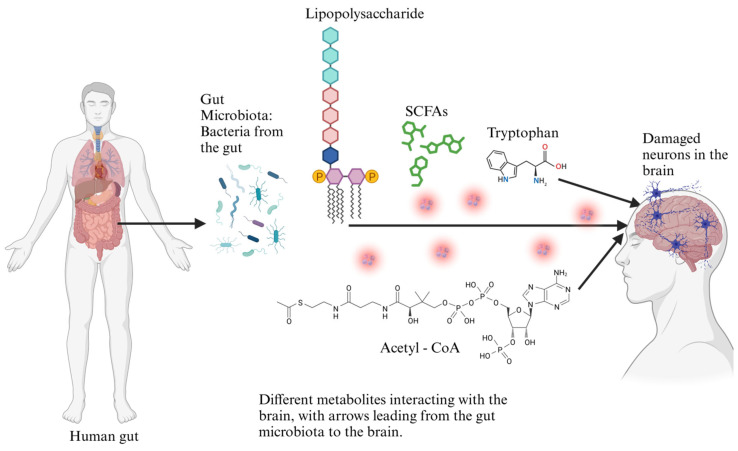
Microbiome metabolites in neurodegeneration (created with BioRender. Busselberg, D. (2025) https://app.biorender.com/illustrations/67ec1664a5549b8cfc5fb58d).

The gut microbiota modulates neuroinflammatory pathways by producing microbial metabolites that influence immune responses. Among these, SCFAs, LPS, and tryptophan metabolites can significantly impact the central nervous system (CNS) by regulating crucial inflammatory mediators such as interleukin-6 (IL-6), tumor necrosis factor-alpha (TNF-α), and nuclear factor kappa B (NF-κB) [40]. These cytokines and transcription factors are central to neuroinflammatory processes and are implicated in the development and advancement of neurodegenerative disorders such as Alzheimer’s and Parkinson’s disease [41]. IL-6 is a pleiotropic cytokine that can exert pro-inflammatory and anti-inflammatory effects depending on the context. Microbial metabolites, particularly LPS from Gram-negative bacteria, can trigger IL-6 secretion by activating toll-like receptor 4 (TLR4) signaling in microglia and astrocytes [42]. This activation contributes to neuroinflammation, exacerbating neuronal damage and synaptic dysfunction. Similarly, SCFAs such as butyrate and propionate can modulate IL-6 expression, either dampening or amplifying inflammation based on their concentration and the host’s immune status [43]. TNF-α, another vital inflammatory cytokine, is upregulated in response to microbial-derived molecules, particularly in conditions of gut dysbiosis. Dysregulated TNF-α levels contribute to the BBB, allowing peripheral immune cells to pass through due to increased permeability and inflammatory mediators to infiltrate the CNS [44]. This cascade can amplify neuroinflammation and neuronal injury, further linking gut microbial composition to neurodegenerative disease progression. NF-κB is a master DNA-binding protein that orchestrates inflammatory responses and is activated by microbial signals. LPS and specific bacterial metabolites can stimulate NF-κB pathways via TLR signaling, producing IL-6, TNF-α, and other pro-inflammatory cytokines [45]. Chronic NF-κB activation in glial cells sustains neuroinflammation and contributes to neurodegeneration by promoting oxidative stress and apoptosis. However, specific microbial metabolites, such as indole derivatives from tryptophan metabolism, can counteract NF-κB activation and provide neuroprotective effects [46]. The interplay between gut microbial metabolites and neuroinflammatory mediators indicates the critical role of the gut–brain axis in neurological health. The realization of these molecular crosstalk mechanisms could pave the way for microbiome-targeted therapies aimed at modulating neuroinflammation and mitigating neurodegenerative diseases [47]. As shown in Table 1, primary microbiota-derived metabolic byproducts, including SCFAs, lipopolysaccharides, and tryptophan metabolites, play significant roles in modulating neuroinflammation and influencing the progression of neurodegenerative diseases. Microbiome-derived metabolites are essential for neurodegeneration through modulation of neuroinflammatory pathways and neurotransmitter balance (Figure 2).

## 3. Inflammatory and Neuroimmune Modulation by Gut Microbiota

### 3.1. Gut Microbiota and the Blood–Brain Barrier (BBB)

The BBB serves as a critical defense mechanism, regulating the passage of substances between the bloodstream and the CNS. It comprises endothelial cells that form tight junctions, protecting the brain from potentially harmful substances, including pathogens and toxins [61]. Recent research has uncovered a complex relationship between the microbiota of the gut and the BBB, with gut-derived metabolites playing a significant role in maintaining or disrupting BBB integrity. The microbiota produces various metabolites, such as SCFAs, tryptophan derivatives, and bile acids, which can influence endothelial cell function and modulate the permeability of the BBB [21]. SCFAs, especially butyrate, have been shown to promote the expression of tight junction proteins in endothelial cells, thereby enhancing BBB integrity and protecting the brain from inflammatory insults. On the other hand, dysbiosis, or an imbalance in the gut microbiota, can disrupt this delicate equilibrium and negatively affect BBB function [62]. When the gut microbiota is disrupted, the production of beneficial metabolites like SCFAs can decrease, while the abundance of harmful microbial components such as LPS can increase. LPS, a potent endotoxin derived from the outer membrane of Gram-negative bacteria, can enter the bloodstream when the intestinal barrier is compromised [63]. Gut dysbiosis is often triggered by factors such as aging, a Western-style diet, or the overuse of antibiotics. This reduces beneficial SCFAs like butyrate, which are critical for maintaining tight junction integrity within the BBB [64]. It is essential to distinguish between the structural role of tight junction proteins in maintaining the physical barrier of the BBB and the functional role of microbial metabolites like SCFAs and LPS in modulating its permeability and immune response [65]. A healthy gut microbiota supports BBB tight junctions through SCFA production. Still, when dysbiosis occurs, the resulting decrease in SCFAs is coupled with an increase in harmful microbial components, particularly lipopolysaccharides (LPS) [66]. Once in the bloodstream, LPS triggers systemic inflammation and contributes to endothelial dysfunction by upregulating inflammatory cytokines such as interleukin-6 (IL-6) and tumor necrosis factor-alpha (TNF-α). This inflammatory state increases BBB permeability, allowing peripheral immune cells and neurotoxic molecules to infiltrate the brain [67].

The sequence typically follows this cascade: gut dysbiosis → ↑ LPS and inflammatory cytokines → BBB disruption → neuroinflammation [26].

Once in circulation, LPS triggers systemic inflammation and can directly affect the BBB by increasing its permeability. This compromised barrier allows immune cells and inflammatory mediators to infiltrate the brain, promoting neuroinflammation and contributing to the development and progression of neurodegenerative diseases [65]. Increased BBB permeability due to dysbiosis leads to a cascade of events that exacerbate neuroinflammation. The infiltration of peripheral immune cells, including macrophages and T-cells, into the brain triggers the activation of the CNS-resident immune cells known as microglia [68]. Once activated, the release of pro-inflammatory cytokines, including IL-6 and TNF-α, by microglia amplifies the brain’s inflammatory response. This inflammatory cascade is detrimental to neuronal health and can accelerate neurodegenerative processes, as seen in disorders like Alzheimer’s and Parkinson’s diseases [69]. A leaky BBB allows the entry of neurotoxic molecules, including amyloid-beta, which can directly promote neuronal damage and synaptic dysfunction. Overall, gut microbiota plays a crucial role in regulating the integrity of the BBB [70]. A balanced microbiota helps maintain a robust BBB, protecting the brain from external insults. At the same time, dysbiosis disrupts this protective barrier, leading to increased permeability, neuroinflammation, and an elevated risk of neurodegeneration [71]. Interpreting the mechanisms by which gut microbiota influence BBB function opens the door for potential therapeutic strategies to restore gut health and prevent or mitigate the detrimental effects of BBB dysfunction in neurodegenerative diseases.

### 3.2. Chronic Inflammation as a Driving Force of Neurodegeneration

**Figure 3:**
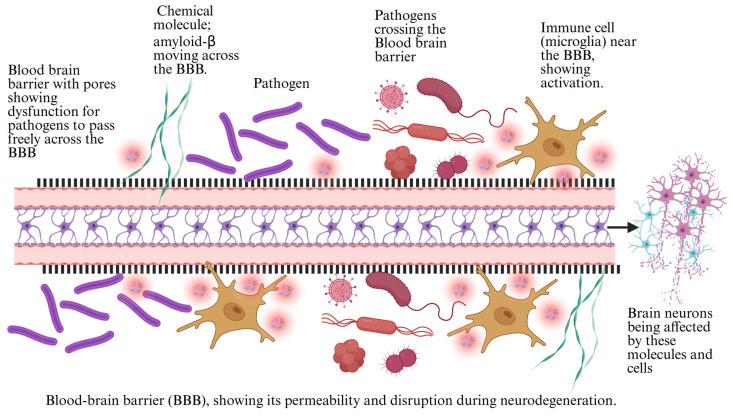
Mechanisms of BBB dysfunction in neurodegeneration (created with BioRender. Busselberg, D. (2025) https://app.biorender.com/illustrations/67ec1ef5d1158b2a397bcc91).

Chronic inflammation is a central factor in the development and progression of neurodegenerative diseases, such as Alzheimer’s, Parkinson’s, and multiple sclerosis. A growing body of evidence suggests that gut microbiota is pivotal in initiating and perpetuating systemic inflammation that impacts the brain [72]. Microbial metabolites derived from gut bacteria can influence immune responses by activating principal inflammatory pathways, including the upregulation of inflammatory cytokines, such as interleukin-6 and TNF-alpha [73]. These cytokines are central to the inflammatory cascade and contribute to neuronal damage, synaptic dysfunction, and the neuroinflammatory environment observed in neurodegenerative diseases. Dysbiosis, or an imbalance in the gut microbiota, can exacerbate this inflammatory response, leading to the activation of chronic inflammation within the CNS [74]. The activation of inflammatory pathways, mainly through IL-6, TNF-α, and nuclear factor kappa B (NF-κB), is closely linked to neurodegenerative processes. IL-6 and TNF-α are potent pro-inflammatory cytokines produced by various immune cells, including microglia and astrocytes, in response to microbial signals [75]. These cytokines can activate NF-κB, a transcription factor that governs the expression of numerous genes involved in inflammation, cell survival, and apoptosis. The continuous stimulation of NF-κB in the brain amplifies neuroinflammation and results in neuronal injury, contributing to the progressive nature of neurodegeneration [76]. Thus, the microbiota-induced activation of these pathways plays a vital role in maintaining the chronic inflammatory state that accelerates neurodegenerative disease progression. One of the most significant microbial factors that drive systemic inflammation is LPS, a component of the outer membrane of Gram-negative bacteria [77]. LPS can enter the bloodstream through an impaired intestinal barrier, particularly in gut dysbiosis. Once in the circulation, LPS acts as a potent endotoxin, triggering systemic inflammation by activating immune cells through toll-like receptor 4 (TLR4). This releases pro-inflammatory cytokines, including IL-6 and TNF-α, which can reach the brain via the bloodstream, exacerbating neuroinflammation [78]. The gut-derived LPS-mediated systemic inflammation is particularly relevant in neurodegenerative diseases, as it has been shown to increase BBB permeability, permitting the entry of immune cells and inflammatory mediators to penetrate the brain more efficiently [79]. This breakdown of the BBB facilitates further neuroinflammation and neuronal damage, reinforcing the vicious cycle of chronic inflammation and neurodegeneration. Chronic inflammation, driven by microbiota-derived factors such as IL-6, TNF-α, and LPS, is a leading mechanism in the pathogenesis of neurodegenerative diseases [80]. The gut microbiota influences local gut immune responses and profoundly affects the central nervous system, exacerbating neuroinflammation and accelerating neurodegeneration. Recognizing these mechanisms paves the way for new therapeutic approaches to modulate the microbiota to reduce chronic inflammation and its detrimental effects on brain health [81]. BBB dysfunction is a critical factor in neurodegeneration, influenced by inflammatory cytokines, microbial metabolites, and oxidative stress (Figure 3).

## 4. Role of Diet in Modulating Gut Microbiota and Neurodegeneration

### 4.1. Dietary Patterns and Their Effects on the Gut–Brain Axis

Diet plays a pivotal role in regulating the microbiota–brain interaction, the exchange network across the gut–brain interface, influencing cognitive function, mood, and even neurodegenerative disease emergence. The composition of the diet can profoundly impact the gut microbiota, which in turn affects brain health [82]. Two commonly studied dietary patterns are the Western and Mediterranean diets, each of which has distinct effects on gut microbiota and cognitive function. The Western diet, characterized by a high intake of processed foods, sugars, unhealthy fats, and low fiber, has been associated with detrimental effects on gut health and cognitive function [83]. This dietary pattern promotes the growth of pathogenic bacteria in the gut, leading to a disruption of the microflora, or dysbiosis, which is associated with increased inflammation and impaired brain health. Research suggests that individuals who adhere to a Western diet can experience accelerated cognitive decline and a higher risk of developing neurodegenerative diseases such as Alzheimer’s and Parkinson’s [84]. The high levels of processed sugars and fats in this diet contribute to an inflammatory environment in the gut and the brain, promoting neuroinflammation and altering brain function [85]. In contrast, the Mediterranean diet, rich in fruits, vegetables, yam [86], whole grains, legumes, healthy fats (mainly olive oil), lean proteins, and pepper soup [87], has been consistently associated with better cognitive function and a reduced risk of neurodegenerative diseases [88]. This diet promotes a more diverse and balanced gut microbiota, supporting the growth of beneficial bacteria that produce metabolites like SCFAs, which are known for their anti-inflammatory and neuroprotective properties [89]. The Mediterranean diet’s emphasis on polyphenols, antioxidants, and healthy fats helps reduce oxidative stress and inflammation in the brain, contributing to improved cognitive health. Studies have shown that individuals following a Mediterranean diet experience slower cognitive decline and demonstrate better cognitive performance in aging [90]. The diet’s positive effects on intestinal microbes and their anti-inflammatory properties are critical in enhancing cognitive function and protecting the brain from age-related neurodegenerative diseases [91]. Furthermore, high-fat, low-fiber diets, often a hallmark of the Western diet, have been shown to promote microbiota dysbiosis. These diets foster the overgrowth of Firmicutes bacteria, which are associated with inflammation and an imbalance in gut health, while decreasing the concentration of Bacteroidetes in fiber fermentation and SCFA production [92]. Reduction in fiber intake in high-fat, low-fiber diets directly impacts SCFA synthesis, which is essential to support gut barrier integrity and regulate immune responses. The imbalance between beneficial and pathogenic microbes leads to increased gut permeability, or “leaky gut”, which facilitates the translocation of harmful bacterial products like LPS into the bloodstream [93]. These microbial products can trigger systemic inflammation, subsequently impacting the brain and leading to neuroinflammation and neuronal damage. This inflammation in the brain is a chief factor during the onset of neurodegenerative diseases [94]. Therefore, the lack of fiber and the dominance of unhealthy fats in high-fat diets exacerbate microbiota dysbiosis and contribute to cognitive decline and brain dysfunction. Dietary patterns, particularly the Western and Mediterranean diets, significantly influence the gut–brain axis and cognitive function [95]. The typical Western diet, rich in unhealthy fats and sugars and low in fiber, fosters microbiota dysbiosis, leading to neuroinflammation and cognitive decline. In contrast, the Mediterranean diet promotes a healthy, balanced microbiota and has neuroprotective effects, supporting cognitive function and reducing the risk of neurodegenerative diseases [96]. These findings mark the importance of dietary choices in maintaining gut health and brain function, indicating that nutritional interventions might be decisive in preventing neurodegeneration.

### 4.2. Bioactive Dietary Compounds with Neuroprotective Potential

Bioactive dietary compounds in various foods have garnered increasing attention for their potential neuroprotective effects. These compounds can influence brain health through multiple mechanisms, including anti-inflammatory properties and modulation of the gut microbiota [97]. Among these bioactive compounds [98], polyphenols and omega-3 fatty acids stand out for their promising roles in neuroprotection. Polyphenols, such as resveratrol and curcumin, are widely recognized for their powerful antioxidant and anti-inflammatory effects, which can help protect the brain from neurodegenerative diseases and cognitive decline [99]. Resveratrol, a polyphenol found in red wine, grapes, and berries, has been shown to exert anti-inflammatory effects by modulating several signaling pathways involved in inflammation. It inhibits the activation of pro-inflammatory cytokines and enzymes, such as TNF-α and COX-2, which play a central role in the inflammatory response in the brain [100]. Resveratrol also activates the SIRT1 pathway, which has been associated with cellular stress resistance and promoting neuronal survival. These effects contribute to reducing neuroinflammation, which is a hallmark of many neurodegenerative conditions, including Alzheimer’s and Parkinson’s diseases [101]. Similarly, curcumin, the active compound in turmeric, has been extensively studied for its anti-inflammatory and antioxidant properties. Curcumin can cross the blood-brain barrier and suppress the activation of microglia, the brain’s innate immune cells, thus preventing excessive inflammation in the CNS [102]. It also reduces oxidative stress by scavenging free radicals and promoting the expression of antioxidant enzymes. Both resveratrol and curcumin are potent modulators of inflammatory pathways, making them valuable dietary components for reducing neuroinflammation and supporting brain health. Omega-3 fatty acids, particularly those found in fatty fish, flaxseeds, and walnuts, are another group of bioactive compounds with significant neuroprotective potential [103]. Omega-3 fatty acids, including EPA (eicosapentaenoic acid) and DHA (docosahexaenoic acid), have been shown to exert neuroprotective actions through various pathways, including the regulation of the gut flora [104]. These fatty acids are critical components of neuronal membranes and are vital in maintaining brain structure and function. Omega-3 fatty acids also exhibit anti-inflammatory activity, helping to decrease the activation of cytokines that induce inflammation and immune cells in the brain [105]. Furthermore, omega-3 fatty acids promote the growth and function of beneficial gut bacteria, which can positively influence the gut–brain axis. By enhancing the diversity of the gut microbiota and supporting the production of anti-inflammatory metabolites like SCFAs, omega-3 fatty acids help to maintain gut health, reduce systemic inflammation, and protect the brain from neurodegenerative damage [106]. In addition to their direct effects on brain health, both polyphenols and omega-3 fatty acids have the potential to influence the gut microbiota, which is increasingly recognized as a crucial mediator of brain function. The modulation of gut microbiota composition through diet can impact the synthesis of neuroactive metabolic products, the control of inflammation, and the integrity of the blood–brain barrier [107]. In this way, these bioactive compounds protect the brain through their inflammation-reducing and antioxidant properties and help maintain the delicate balance of the gut–brain axis. As such, including polyphenols, like resveratrol and curcumin, and omega-3 fatty acids in the diet could be a powerful strategy for preventing or mitigating neurodegenerative diseases and supporting overall cognitive health [108]. Bioactive dietary compounds such as polyphenols (resveratrol and curcumin) and omega-3 fatty acids possess significant neuroprotective potential [109]. These compounds exert their effects through anti-inflammatory and antioxidant mechanisms and by modulating the gut microbiota [110]. By incorporating these compounds into their diet, individuals can support brain health, reduce neuroinflammation, and protect against the onset of neurodegenerative diseases.

### 4.3. Clinical Relevance: Can Diet-Based Modulation of Gut Microbiota Slow Alzheimer’s Progression?

The role of diet in modulating the gut microbiota and its potential to slow the progression of Alzheimer’s disease has been the focus of several human trials in recent years. These studies aim to determine whether specific dietary patterns or bioactive compounds can positively influence cognitive function and delay the onset or progression of Alzheimer’s by affecting the gut–brain axis [111]. Evidence from human trials suggests that dietary interventions can significantly impact cognitive performance and brain health. For instance, the Mediterranean diet, rich in fruits, vegetables, whole grains, and healthy fats, has been shown to reduce the risk of developing Alzheimer’s and slow cognitive decline in aging populations [112]. Several clinical studies have indicated that individuals following this diet exhibit improved cognitive function, reduced neuroinflammation, and a healthier gut microbiota composition compared to Western diets. Similarly, clinical trials examining the effects of specific nutrients, such as omega-3 fatty acids and polyphenols, on Alzheimer’s progression have yielded promising results [113]. Omega-3 supplementation, particularly DHA, is found to enhance cognitive performance and decrease markers of neuroinflammation in elderly individuals at risk for Alzheimer’s. Polyphenols, such as those found in berries, green tea, and turmeric, have also demonstrated potential cognitive benefits, possibly by reducing oxidative stress and inflammation in the brain [114]. These dietary interventions can exert their effects by modulating gut microbiota, which influences systemic inflammation and brain health. Furthermore, a recent systematic review and meta-analysis has strengthened the link between gut microbiota composition and cognitive function in individuals with Alzheimer’s disease, indicating that the gut microbiome may act both as a contributor to disease progression and a promising therapeutic target [115].

### 4.4. Molecular Pathways Linking Diet to Neuroinflammation

**Figure 4:**
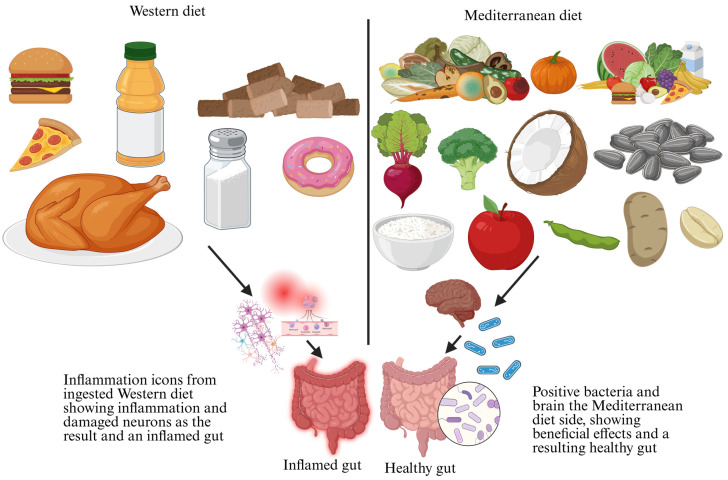
Western vs. Mediterranean diet’s impact on the gut–brain axis (created with BioRender. Busselberg, D. (2025) https://app.biorender.com/illustrations/67ec28ef6304a66d6efa6b7c).

The connection between diet and neuroinflammation is increasingly being understood through molecular mechanisms that link dietary components to cellular and epigenetic processes in the brain. One of the critical pathways through which diet impacts neuroinflammation is the production of SCFAs [116]. SCFAs, such as butyrate, propionate, and acetate, are produced by the fermentation of dietary fiber by gut bacteria and have been shown to play a crucial role in regulating the immune system and modulating inflammation. SCFAs can enter the bloodstream and reach the brain, where neuroinflammatory pathways are influenced by activating specific receptors, such as G-protein-coupled receptors (GPCRs) [22]. One of the most notable effects of SCFAs is their ability to modulate gene expression through epigenetic mechanisms. SCFAs can inhibit histone deacetylases (HDACs), enzymes involved in chromatin remodeling, which leads to activating anti-inflammatory genes [117]. This epigenetic modulation helps to reduce neuroinflammation in the brain, a significant driver of neurodegenerative diseases such as Alzheimer’s and Parkinson’s. By promoting the expression of anti-inflammatory cytokines and reducing the levels of pro-inflammatory mediators, SCFAs contribute to a neuroprotective environment that can help slow the progression of these diseases [118]. Another molecular pathway linking diet to neuroinflammation involves polyphenols, bioactive compounds in plant-based foods like fruits, vegetables, and tea. Polyphenols, such as curcumin and resveratrol, have been shown to modulate neuroinflammation by influencing autophagy in neuronal cells [119]. Autophagy is a cellular process that removes damaged or dysfunctional components from cells, including misfolded proteins implicated in neurodegenerative diseases [120]. Polyphenols can stimulate autophagy by activating essential signaling pathways, such as the AMPK-mTOR pathway, which enhances the degradation of damaged proteins and reduces cellular stress. This process protects neurons from oxidative damage and helps to maintain neuronal homeostasis and function [121]. In neuroinflammation, polyphenol-induced autophagy can also promote the clearance of neurotoxic substances and inflammatory cytokines, further reducing inflammation and supporting brain health. By influencing both epigenetic mechanisms through SCFAs and cellular processes such as autophagy through polyphenols, diet can play a pivotal role in mitigating neuroinflammation and offering neuroprotective effects against advancing neurodegenerative disorders [122]. Diet is critical in modulating neuroinflammation through various molecular pathways. SCFAs synthesized by gut microbiota from dietary fiber fermentation regulate immune responses and gene expression via epigenetic modulation, contributing to a reduction in neuroinflammation [123]. Conversely, polyphenols influence neuronal autophagy, promoting the clearance of damaged proteins and reducing oxidative stress. These dietary mechanisms support brain health and offer promising strategies for slowing the progression of neurodegenerative diseases, such as Alzheimer’s and Parkinson’s, by targeting the underlying processes of neuroinflammation [124]. As shown in Table 2, dietary components can significantly affect the intestinal microbial community, influencing the growth of microflora and the balance of microbial diversity. Dietary patterns significantly influence the gut–brain axis, with the Western diet exacerbating neuroinflammation, while the Mediterranean diet promotes neuroprotection (Figure 4).

## 5. Mechanistic Insights: Gut Microbiota in Amyloid-β and Tau Pathology

### 5.1. Dysbiosis and Tau Hyperphosphorylation

Dysbiosis, a disruption in the balance of the gut microbiota, has been increasingly associated with the pathogenesis of Alzheimer’s disease, particularly concerning tau pathology. Tau proteins are critical for maintaining the structure of neurons, but in neurodegenerative diseases like Alzheimer’s, tau can become hyperphosphorylated, forming abnormal protein aggregates in the brain [134]. These abnormal proteins disrupt neuronal activity and play a role in cognitive decline. One main factor contributing to tau hyperphosphorylation is bacterial endotoxins, such as LPS, which are released by pathogenic bacteria in the gut. When the gut microbiota becomes dysbiotic, the overgrowth of harmful bacteria can lead to the increased release of LPS into the bloodstream, a condition known as endotoxemia [135]. LPS can cross the blood–brain barrier, triggering inflammation and oxidative stress in the brain. This inflammation can activate several signaling pathways that contribute to tau hyperphosphorylation, including the activation of kinases such as glycogen synthase kinase 3 beta (GSK-3β) and cyclin-dependent kinase 5 (CDK5), which are involved in tau phosphorylation [136]. Oxidative stress induced by LPS can produce reactive oxygen species (ROS), further exacerbating tau pathology and promoting neurofibrillary tangles’ formation. The relationship between gut dysbiosis, bacterial endotoxins, and tau hyperphosphorylation conveys the importance of maintaining a balanced gut microbiota to protect against neurodegenerative diseases [137]. Handling dysbiosis through dietary interventions or probiotic supplementation can help reduce endotoxin release and oxidative stress, potentially mitigating tau pathology and slowing the progression of Alzheimer’s disease. Both SCFAs, particularly butyrate, and gut dysbiosis play significant roles in the pathophysiology of Alzheimer’s [138]. Butyrate helps to activate microglia, facilitating the clearance of amyloid-β plaques. At the same time, dysbiosis and the release of bacterial endotoxins like LPS contribute to tau hyperphosphorylation and the progression of neurodegeneration [139]. These findings highlight the critical connection between gut health, microbiota composition, and brain function, emphasizing the potential for dietary interventions to modulate the connection between the gut and brain, and provide treatment benefits for conditions related to neurodegenerative diseases.

### 5.2. Neurotransmitter Metabolism by Gut Microbiota

The gut microbiota is crucial in metabolizing various neurotransmitters, which are essential for maintaining brain function and regulating mood, cognition, and behavior. Among the neurotransmitters influenced by gut microbiota are serotonin, dopamine, and acetylcholine, all of which are involved in neuropsychiatric disorders such as depression, anxiety, and Parkinson’s disease [140]. The gut is a primary site for synthesizing serotonin, with approximately 90–95% of the body’s serotonin produced in the gastrointestinal tract. Microbial populations within the gut can influence serotonin levels by affecting the synthesis and breakdown of this neurotransmitter [141]. Certain gut bacteria, such as *Lactobacillus* sp. and *Bifidobacterium* sp., have been shown to enhance serotonin production through the action of specific enzymes. By modulating its absorption and metabolism, gut bacteria can influence the availability of tryptophan, the precursor for serotonin synthesis [33]. The gut microbiota’s modulation of serotonin levels can directly impact brain serotonin signaling, influencing mood and behavior. Similarly, the gut microbiome influences dopamine metabolism, which plays a pivotal role in motivation, reward, and motor control [142]. Particular bacterial species in the gut, such as Bacteroides, can influence the production of dopamine precursors, which can subsequently impact dopamine signaling in the brain. In Parkinson’s disease, a condition characterized by the degeneration of dopamine-producing neurons in the brain, the gut microbiota has been implicated in regulating dopaminergic signaling and modulating neuroinflammation [143]. Furthermore, gut microbiota influences acetylcholine metabolism, a neurotransmitter vital to learning, memory, and muscle function. Research suggests that gut bacteria can modulate choline levels, the precursor for acetylcholine, thereby affecting its synthesis [144]. Dysbiosis, or imbalances in the gut microbiota, can lead to altered neurotransmitter metabolism, contributing to various neurological and psychiatric disorders [145]. Therefore, appreciating the intricate relationship between the gut microbiota and neurotransmitter metabolism is crucial for exploring potential therapeutic approaches to modulate brain function and treat diseases like depression, Parkinson’s disease, and Alzheimer’s.

### 5.3. Role of the Gut Microbiota in Misfolded Protein Clearance

**Figure 5:**
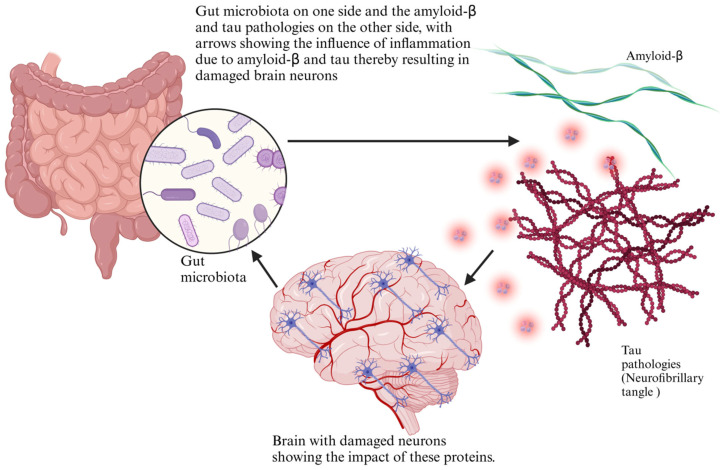
Amyloid-β and tau pathology modulation by gut microbiota (created with BioRender. Busselberg, D. (2025) https://app.biorender.com/illustrations/67ec33dab450d927accb02f8).

The gut microbiota is increasingly recognized for regulating cellular processes beyond the gastrointestinal system, including clearing misfolded proteins that accumulate in neurodegenerative diseases. Misfolded proteins, such as amyloid-β in Alzheimer’s and α-synuclein in Parkinson’s disease, can aggregate and form toxic plaques that disrupt cellular function and contribute to neurodegeneration [146]. One of the mechanisms by which the body clears these misfolded proteins is through the proteasomal degradation system and autophagy, both of which are critical cellular processes for maintaining protein homeostasis. The gut microbiota can influence these processes by modulating systemic inflammation, oxidative stress, and the production of metabolites that directly impact cellular pathways involved in protein degradation [147]. Proteasomal degradation, a process in which damaged or misfolded proteins are tagged for destruction by ubiquitin and then degraded by the proteasome, may be modulated by microbial metabolites like SCFAs. SCFAs, including butyrate, have been shown to promote proteasomal activity, thereby enhancing the clearance of misfolded proteins [148]. Autophagy, a cellular process encompassing the intake and degradation of misfolded proteins within lysosomes, is also influenced by the gut microbiota. Particular bacterial species can stimulate autophagy by producing metabolites such as SCFAs, which activate major signaling pathways such as the mTOR and AMPK pathways [149]. By promoting autophagic clearance, the gut microbiota helps reduce the accumulation of toxic misfolded proteins and mitigates the neurodegenerative processes associated with diseases like Alzheimer’s and Parkinson’s. Moreover, the gut microbiome can influence the immune system, which plays a significant role in the clearance of misfolded proteins [150]. Dysbiosis, a disruption in gut microbial balance, can result in chronic low-grade immune response and the activation of the brain’s immune cells, the microglia. When microglia are overactivated, neuroinflammation occurs, hindering the clearance of misfolded proteins [151]. Thus, a balanced gut microbiota that promotes anti-inflammatory pathways can support the brain’s ability to clear toxic protein aggregates, reducing the risk of neurodegenerative processes. The role of the gut microbiota in influencing proteasomal degradation, autophagy, and immune modulation highlights its essential role in maintaining protein homeostasis and protecting the brain from accumulating misfolded proteins contributing to neurodegenerative diseases [152]. Through dietary interventions or probiotic supplementation aimed at restoring gut health, it is possible to enhance these cellular clearance processes and slow the progression of neurodegeneration. As shown in Table 3, several clinical trials have investigated diet–microbiota interventions in Alzheimer’s, exploring the impacts of various eating habits, supplements, as well as probiotics on mental function and gut health. Gut microbiota play a pivotal role in modulating amyloid-β aggregation and tau pathology, influencing neurodegenerative progression through microbial metabolites and immune signaling (Figure 5).

### 5.4. Molecular Targets of Microbial Metabolites in Neuroprotection

Microbial metabolites exert neuroprotective effects by interacting with various molecular targets and signaling pathways involved in neuroinflammation, neuronal survival, and cognitive function [20]. Short-chain fatty acids (SCFAs) such as butyrate, acetate, and propionate influence inflammation, intestinal barrier integrity, and the sympathetic nervous system by activating G-protein-coupled receptors like GPR41 and GPR43, which play a significant role in neurodegeneration [159]. Butyrate inhibits histone deacetylases (HDACs), leading to epigenetic changes that promote anti-inflammatory gene expression and support neuronal plasticity. This process enhances the production of brain-derived neurotrophic factor (BDNF), which is crucial for cognitive function [160]. Bile acids activate the farnesoid X receptor (FXR) and TGR5, which regulate both metabolic and immune responses, helping to reduce neuroinflammation and improve neuronal health [161]. Tryptophan-derived metabolites from the gut microbiota engage the aryl hydrocarbon receptor (AhR), impacting immune responses and oxidative stress. Some of these metabolites also contribute to serotonin production, linking microbiota activity to neurotransmitter balance and mood regulation [33]. These molecular mechanisms provide potential targets for microbiome-based therapies that could slow or prevent neurodegenerative diseases, such as Alzheimer’s disease.

## 6. Future Directions: Microbiome-Based Therapeutic Interventions

### 6.1. Probiotics, Prebiotics, and Postbiotics for Neuroprotection

Probiotics, prebiotics, and postbiotics have garnered attention for their potential neuroprotective effects, particularly concerning neurodegenerative conditions like Alzheimer’s disease. Probiotics, which are live beneficial bacteria, are crucial in maintaining gut health and influencing the gut–brain axis [162]. Certain strains of probiotics have been shown to have direct neuroprotective effects. For instance, *Lactobacillus* sp. and *Bifidobacterium* species are among the most studied probiotics positively affecting brain health. These strains can modulate the immune system, reduce gut inflammation, and influence the synthesis of neuroactive compounds, including serotonin [163]. These probiotics have been shown to enhance the integrity of the blood–brain barrier, which can help protect the brain from neuroinflammation and oxidative damage. The neuroprotective effects of probiotics are thought to be mediated through their ability to modulate gut microbiota composition, thereby improving gut health and reducing systemic inflammation, contributing to cognitive function and neuroprotection [164]. Prebiotics, which are food components that resist digestion and support the proliferation and function of beneficial gut microbes, also play a significant role in neuroprotection. One of the core benefits of prebiotics is their ability to enhance the production of SCFAs like butyrate [165]. SCFAs are manufactured by gut bacteria during the breakdown of dietary fibers through fermentation and are known to have multiple beneficial effects on brain health. Butyrate, in particular, has neuroprotective properties because of its capacity to reduce neuroinflammation, enhance mitochondrial function, and promote the removal of harmful proteins, like amyloid-β [166]. SCFAs can improve cognitive function by influencing gene expression in the brain through epigenetic modifications, such as histone acetylation. Together, probiotics and prebiotics present a promising approach to improving mental health in potentially slowing the progression of neurodegenerative diseases like Alzheimer’s [167]. Postbiotics, the bioactive compounds produced by probiotics during fermentation, have also been shown to have neuroprotective effects. These include metabolites such as SCFAs, antimicrobial peptides, and other signaling molecules that can modulate inflammation, promote neuronal survival, and enhance cognitive function [168]. By acting on the gut and the brain, postbiotics offer a novel and complementary approach to supporting brain health. Emerging research suggests that postbiotics could be combined with probiotics and prebiotics to optimize neuroprotective outcomes, providing a multi-faceted strategy for managing neurodegenerative diseases.

### 6.2. Fecal Microbiota Transplantation (FMT) as a Potential Therapy

FMT is a procedure that involves transferring gut microbiota from a healthy donor into a recipient, is gaining attention as a potential therapeutic approach for Alzheimer’s disease and other neurodegenerative conditions. The goal of FMT is to restore a healthy microbiome in patients with dysbiosis, which has been linked to several chronic diseases, including neurodegenerative disorders [169]. For Alzheimer’s disease, the idea is that restoring a balanced gut microbiota could improve cognitive function, reduce neuroinflammation, and slow disease progression. Preclinical studies in animal models have shown that FMT can have beneficial effects on brain health, including improved memory, reduced amyloid plaque accumulation, and decreased neuroinflammation [170]. Additional research has shown that FMT can enhance cognitive function in animal models by modulating the gut–brain axis, which influences neuroinflammation and synaptic plasticity [171]. Butyrate, a short-chain fatty acid produced by gut microbiota during the fermentation of dietary fiber, plays a key role in maintaining brain health. It regulates microglial activity, which is crucial for managing neuroinflammation [172]. Preclinical studies indicate that butyrate inhibits histone deacetylases (HDAC), triggering changes in gene expression that foster a neuroprotective environment. Through HDAC inhibition, butyrate promotes histone acetylation, leading to the activation of anti-inflammatory genes and the suppression of pro-inflammatory cytokines in microglia, thus reducing neuroinflammation and improving cognitive function [173]. These mechanisms are extensively documented in studies exploring the relationship between gut-derived metabolites and brain function, particularly in Alzheimer’s disease and other neurodegenerative diseases.

These findings have led researchers to hypothesize that FMT could be a viable treatment for Alzheimer’s patients by promoting a healthier gut microbiome that positively influences the brain. The potential of FMT as a therapy for Alzheimer’s disease is based on the concept that gut microbiota imbalances contribute to neuroinflammation and the progression of amyloid-β and tau pathologies, both of which are central to Alzheimer’s [174]. By restoring the gut microbiota, FMT could reduce systemic inflammation and promote a healthier environment for neuronal function. Several clinical trials are currently investigating the effectiveness of FMT in individuals with Alzheimer’s disease, focusing on outcomes such as cognitive improvement, gut microbiota composition, and biomarkers of neuroinflammation [175]. However, while early studies show promise, the clinical application of FMT for Alzheimer’s is still in its infancy. There are several difficulties to consider, including identifying appropriate donor microbiota, the standardization of procedures, and the long-term safety and efficacy of the treatment [176]. Despite these obstacles, FMT remains an exciting potential therapy that could help modulate the gut–brain axis and provide a novel approach to treating Alzheimer’s disease and similar neurodegenerative conditions.

Fecal microbiota transplantation (FMT) presents promising therapeutic potential for neurodegenerative disorders, as several ethical and logistical challenges must be resolved before it can be widely implemented [177]. One of the primary concerns is the absence of standardized protocols, which differ across institutions and regions, hindering consistent evaluation of safety and efficacy [178]. Despite comprehensive donor screening, there remains a risk of transmitting pathogenic or unidentified harmful microbes, which is an issue of particular concern in immunocompromised populations such as elderly patients with Alzheimer’s disease [179]. Patient acceptance is another critical factor, influenced by cultural norms and psychological discomfort associated with the nature of the procedure. Many individuals may view FMT as invasive or unappealing, leading to hesitation or outright refusal. These perceptions highlight the importance of thorough patient education and transparent informed consent processes [180]. In addition, regulatory ambiguity continues to challenge the integration of FMT into clinical practice. As FMT straddles the domains of biologics and procedural medicine, regulatory agencies are still working to define appropriate oversight mechanisms, particularly for use in high-risk or vulnerable patient groups [181]. While FMT holds considerable promise as a novel intervention for neurodegenerative diseases, its successful adoption depends on establishing standardized, safe procedures, more straightforward regulatory guidelines, and ethically sensitive strategies to improve patient understanding and acceptance.

A critical evaluation of FMT’s current experimental landscape reveals considerable variability in outcomes across clinical trials, raising concerns about the reproducibility of results and the influence of patient-specific factors [182]. The absence of standardized criteria for donor selection and microbiota profiling further complicates inter-study comparisons. The potential risks, such as adverse effects linked to the microbiome, long-term immune consequences, and microbiota instability, must not be overlooked. These concerns are especially pertinent in older populations, where immune aging and comorbidities may heighten the risks [183]. As such, there is a pressing need to establish standardized FMT protocols and robust regulatory oversight. Ethical considerations should also be given priority, including clear communication of potential risks, equitable treatment access, and careful patient selection to ensure safety and effectiveness, particularly in vulnerable groups like the elderly [184].

### 6.3. Personalized Microbiome-Based Dietary Strategies

Personalized microbiome-based dietary strategies represent a promising approach to managing neurodegenerative diseases, including Alzheimer’s. These strategies are rooted in precision nutrition, where dietary recommendations are tailored based on an individual’s unique microbiome profile [185]. Advances in microbiome sequencing technology have made it possible to identify the specific composition and functional characteristics of an individual’s gut microbiota. This allows for a more targeted approach to diet and supplementation, resolving the unique microbial imbalances that can contribute to neuroinflammation and cognitive decline [186]. For instance, individuals with a gut microbiome that is low in probiotic bacteria, such as *Bifidobacterium* sp. and *Lactobacillus* sp., can benefit from dietary interventions that promote the growth of these microbes, such as increasing the intake of prebiotic fibers like inulin or fructooligosaccharides [187]. In contrast, individuals with an overgrowth of pro-inflammatory bacteria can benefit from diets focusing on anti-inflammatory foods, such as polyphenol-rich fruits and vegetables, to help restore balance in the microbiota and reduce neuroinflammation. By focusing on the gut–brain axis, personalized microbiome-based dietary strategies aim to optimize brain health by improving gut health [188]. This approach could help slow the progression of cognitive decline and potentially prevent neurodegeneration. For example, specific microbiome profiles have been associated with higher production of SCFAs, which are known to have neuroprotective effects. In contrast, others can be linked to the increased production of harmful metabolites that exacerbate neuroinflammation [189]. Personalized nutrition can, therefore, guide dietary changes that support healthy microbiota, promoting the production of beneficial metabolites like SCFAs and reducing the production of harmful substances. As research in this field continues to evolve, microbiome-based dietary strategies can become crucial in managing Alzheimer’s and other neurodegenerative diseases, offering a tailored, evidence-based approach to improving cognitive function [190].

### 6.4. Emerging Therapies Modulating the Gut–Brain Link

Emerging therapies targeting the gut–brain connection represent a new frontier for treating neurodegenerative diseases, with metabolite-based drugs showing particular promise for neuroprotection [191]. One of the main aspects of these therapies is their ability to influence the microbiome, which modulates the brain through various biological processes, like immune modulation, inflammation reduction, and the production of neuroactive metabolites [192]. A growing body of research has shown that specific metabolites produced by gut bacteria, such as SCFAs, indoles, and bile acids, can benefit the brain, promoting neuroprotection, reducing neuroinflammation, and enhancing neuronal function [193]. For example, SCFAs like butyrate have been shown to support the integrity of the blood–brain barrier, modulate microglial activity, and protect against neurodegeneration by reducing the accumulation of toxic proteins like amyloid-β. Metabolite-based drugs are being developed to harness the therapeutic potential of these gut-derived compounds [194]. These drugs aim to either directly supply beneficial metabolites or promote their production through modulation of the gut microbiota [195]. For instance, drugs that increase the production of SCFAs in the gut could provide neuroprotective benefits by enhancing the anti-inflammatory effects of these metabolites on the brain [196]. Researchers are exploring probiotics, prebiotics, and postbiotics as adjunct therapies to improve gut health and, by extension, brain health. In particular, therapies that modulate the gut microbiota to reduce systemic inflammation and promote the production of neuroprotective metabolites offer a promising new avenue for treating Alzheimer’s and other neurodegenerative diseases. These emerging therapies are still in the early stages of development but hold significant potential for providing more targeted and effective treatments for neurodegeneration. Focusing on the gut–brain axis and leveraging the power of microbiome-derived metabolites can slow the progression of diseases like Alzheimer’s, improve cognitive function, and enhance overall brain health [197]. As clinical trials continue to explore the efficacy of these metabolite-based therapies, these therapies can offer a novel, more holistic approach to managing neurodegenerative diseases that incorporates both the gut and brain in a therapeutic context. As shown in Table 4, various probiotic and prebiotic strategies have been identified for promoting cognitive health through mechanisms such as modulating gut microbiota, enhancing gut barrier function, and reducing neuroinflammation.

## 7. Complications, Limitations, and Future Research Priorities

### 7.1. Gaps in Understanding the Gut–Brain Axis in Alzheimer’s

While there has been significant progress in the grasp of the gut–brain axis and its implications for Alzheimer’s disease, there are still notable gaps in knowledge. One central area that requires further exploration is the need for large-scale, longitudinal microbiome studies. These studies would help clarify how changes in the gut microbiota over time contribute to the development and progression of Alzheimer’s [206]. Currently, most research is limited to cross-sectional studies, which provide only a snapshot of microbiota composition at a single point. Long-term studies are necessary to establish causal relationships between specific microbial populations, their metabolites, and the onset of neurodegenerative diseases [207]. Longitudinal studies could provide insights into how dietary interventions or microbiome-targeting therapies could prevent or slow the progression of Alzheimer’s, offering more personalized approaches to treatment. Another significant issue is knowing the complex, dynamic interactions between the gut microbiota, the immune system, and the brain [208]. While dysbiosis (microbial imbalance) and the resulting systemic inflammation contribute to neurodegeneration, the exact mechanisms remain unclear. More studies are required to pinpoint specific microbial signatures that could serve as biomarkers for early diagnosis and to understand how these microbial changes influence brain pathology [209]. Ultimately, overcoming these gaps in knowledge will pave the way for more targeted and effective interventions that can alter the microbiome to improve brain health and potentially prevent or treat Alzheimer’s.

### 7.2. Variability in Microbiome Response to Dietary Interventions

One major problem in applying diet-based strategies for improving cognitive health is the variability in individual responses to dietary interventions. People have highly personalized gut microbiomes, influenced by genetics, lifestyle, environmental factors, and previous nutritional habits [210]. As a result, the same dietary intervention can affect each individual differently. For example, prebiotic fibers such as inulin or fructooligosaccharides (FOS) can promote the growth of beneficial bacteria in some individuals but not in others, depending on their baseline microbiota composition [211]. This variability complicates the development of one-size-fits-all dietary recommendations for cognitive health. Individual differences in metabolite production, such as SCFAs, can further impact the effectiveness of nutritional interventions. SCFAs, generated by gut bacteria through the fermentation of dietary fibers, have been shown to have neuroprotective effects [212]. However, not everyone produces the same amount of SCFAs, and this variability can influence how much a person benefits from a diet rich in prebiotics. Therefore, precision nutrition, which tailors dietary recommendations based on an individual’s unique microbiome profile, holds promise as a more practical approach for improving cognitive function and potentially slowing the progression of Alzheimer’s [213]. Research into personalized dietary strategies is essential to address this variability and optimize outcomes for individuals with neurodegenerative diseases.

### 7.3. Integrating Microbiome Science with Precision Medicine

Integrating microbiome science with precision medicine holds immense potential for advancing personalized interventions in the treatment of neurodegenerative diseases like Alzheimer’s. The application of multi-omics approaches, which combine genomics, transcriptomics, metabolomics, and proteomics, allows for a good grasp of how the microbiome interacts with various biological systems to influence health [214]. By profiling an individual’s microbiome alongside their genetic, metabolic, and proteomic data, researchers can gain deeper insights into how microbial imbalances affect disease pathways and identify tailored therapeutic strategies. Multi-omics data can also predict individual responses to specific dietary, probiotic, or pharmaceutical interventions, enabling clinicians to design more personalized, effective treatment plans [215]. Personalized interventions informed by multi-omics data could revolutionize how Alzheimer’s can be approached. For example, precision medicine could enable the identification of specific microbial signatures that predispose individuals to Alzheimer’s, allowing for early interventions targeting those microbial populations before neurodegeneration progresses [216]. These approaches could help predict which dietary interventions or probiotic strains would be most beneficial for each individual, optimizing their effects on cognitive function and overall brain health. Integrating microbiome science with precision medicine offers a promising avenue for developing more targeted, individualized therapies that could ultimately improve outcomes for people with neurodegenerative diseases.

### 7.4. Comparative Analysis of Microbiome-Based vs. Conventional Therapies

As interest in microbiome-based therapies grows, it is essential to compare their effectiveness with conventional pharmaceutical interventions, particularly in treating Alzheimer’s. Diet-based strategies, which focus on modulating the gut microbiome through prebiotics, probiotics, and dietary fiber, are gaining attention for their potential to improve cognitive function and reduce neuroinflammation [217]. These strategies aim to restore balance in the microbiome [218], enhance gut–brain signaling, and reduce the systemic inflammation contributing to neurodegeneration. In contrast, conventional pharmaceutical interventions, such as cholinesterase inhibitors and glutamate regulators, primarily target symptoms of Alzheimer’s, such as memory loss and cognitive decline, but often fail to address the underlying causes of the disease [219]. While pharmaceutical treatments provide symptomatic relief, they do not halt or reverse the progression of the disease, and they are often associated with side effects. On the other hand, diet-based strategies that focus on the gut–brain axis offer a more holistic approach, potentially dealing with the root causes of Alzheimer’s by restoring gut health, reducing inflammation, and modulating neuroprotective pathways [220]. However, the effectiveness of diet-based therapies can vary depending on an individual’s unique microbiome composition. More research is needed to determine their long-term efficacy and safety. Comparatively, pharmaceutical therapies offer more immediate and standardized effects. Still, their inability to modify the disease’s progression highlights the need for adjunctive treatments, such as those targeting the microbiome, to enhance overall outcomes [221]. Ultimately, a combination of both microbiome-based and conventional therapies can offer the best approach for managing Alzheimer’s, improving both symptoms and underlying disease processes. As summarized in Table 5, there are several gaps in knowledge regarding the gut–brain connection in Alzheimer’s disease, with future studies focused on recognizing microbiome-drug interactions, identifying microbial biomarkers, and exploring personalized microbiome-based interventions.

Future research should prioritize large-scale, longitudinal human studies to establish clear causal relationships between gut microbial metabolites and neurodegenerative diseases. Overcoming challenges such as individual variability, non-standardized microbiome assessments, and clinical inconsistencies is critical for clinical application. A promising approach involves integrating multi-omics technologies such as metagenomics, metabolomics, and proteomics alongside personalized microbiome profiling, which could facilitate precision-targeted dietary interventions and microbial therapies. Furthermore, gut microbiota diversity is often reduced in Alzheimer’s patients, and nutritional strategies that support microbiota balance may help restore gut health and enhance cognitive function. Identifying specific microbial strains and dietary components with neuroprotective effects could lead to innovative treatments for neurodegenerative diseases.

In our view, well-controlled and long-term randomized controlled trials (RCTs) are crucial for clarifying the effects of microbiome-based interventions compared to conventional treatments. These trials could determine whether such interventions provide synergistic benefits or contribute to improved cognitive health. The neuroprotective roles of specific microbial strains and dietary components, particularly in restoring microbial diversity in Alzheimer’s patients, warrant further investigation. Research focused on diet-driven modulation of the gut microbiota holds considerable potential for slowing the progression of neurodegenerative diseases and enhancing cognitive health. Still, a more in-depth exploration of the underlying mechanisms is necessary to realize these therapeutic possibilities fully.

## 8. Conclusions

A growing body of evidence underscores the central role of the gut–brain axis in the development and progression of neurodegenerative diseases, particularly Alzheimer’s disease. Research increasingly links gut microbiota dysbiosis, a microbial imbalance, to chronic systemic and neuroinflammation, hallmark features of Alzheimer’s. Key microbial metabolites such as short-chain fatty acids (SCFAs) and gut-derived compounds like lipopolysaccharides (LPS) have been implicated in disrupting blood–brain barrier integrity and activating neuroinflammatory pathways. These insights highlight the pivotal influence of gut health on cognitive function and suggest that modulating the gut microbiota could offer a promising therapeutic strategy for neurodegeneration. The interplay among the gut–brain axis, microbial metabolites, and inflammatory processes is a significant theme in understanding Alzheimer’s pathophysiology. Dietary modulation presents a non-invasive and accessible method for influencing gut microbial composition. Approaches such as prebiotics, probiotics, fiber-rich foods, and bioactive compounds, including polyphenols and omega-3 fatty acids, have demonstrated anti-inflammatory and neuroprotective effects. These interventions can restore microbial balance, enhance gut–brain signaling, and potentially slow the progression of cognitive decline, positioning them as promising complementary therapies alongside existing Alzheimer’s treatments. Unlike pharmaceuticals that often focus on symptom management, these microbiome-targeted strategies may tackle root causes such as inflammation and impaired neural communication. As such, dietary modulation emerges as a crucial pillar of gut microbiota-focused therapeutic interventions in neurodegeneration. Despite these advances, several research gaps remain. The mechanisms by which specific microbes and their metabolites influence brain health are not fully understood, and inter-individual variability in microbiome composition presents a challenge to universal application. Standardized dietary interventions and microbiome assessment protocols are urgently needed to ensure reproducibility and clinical reliability. Moreover, current studies often lack consistent endpoints and comprehensive follow-up periods, limiting the generalizability of findings across diverse populations. Future research should prioritize the identification of microbial biomarkers associated with early-stage Alzheimer’s and cognitive resilience, enabling earlier diagnosis and targeted intervention. There is also a critical need for personalized diet-based strategies, tailored to an individual’s microbiome profile, to optimize therapeutic outcomes. Furthermore, integrating multi-omics tools such as metagenomics, metabolomics, and transcriptomics can provide deeper mechanistic insights and improve patient stratification for clinical trials. Large-scale, longitudinal clinical studies are essential to validate microbiome-based therapies’ long-term efficacy and safety, including dietary modulation and fecal microbiota transplantation (FMT). Efforts should also be directed at defining core microbiome signatures linked to treatment responsiveness and investigating potential interactions between microbiota and conventional Alzheimer’s pharmacotherapies. A synthesis of current research reveals that the gut–brain axis, gut microbiota, and dietary modulation are deeply interconnected in shaping neurocognitive outcomes. By advancing precision nutrition and microbiome-targeted therapies, the field is moving toward a more holistic and practical approach to managing Alzheimer’s disease, emphasizing prevention, personalization, and integration with existing medical treatments. This integrated perspective holds promise for transforming Alzheimer’s care by addressing both symptom alleviation and underlying disease mechanisms. Although promising, microbiome-based dietary interventions must address current limitations by implementing standardized protocols and personalized approaches. Ongoing research could pave the way for integrating these strategies into mainstream Alzheimer’s treatment, alongside conventional pharmaceutical options.

## Figures and Tables

**Table 1:** Major microbiota-derived metabolites and their impact on neurodegeneration.

Microbiota-Derived Metabolite	Source/Pathway	Impact on Neurodegeneration	Mechanism of Action
Short-Chain Fatty Acids (SCFAs)	Breakdown of dietary fibers by intestinal microbes	Neuroprotective; reduces neuroinflammation and enhances BBB integrity	SCFAs, particularly butyrate, regulate histone deacetylases (HDACs), promoting anti-inflammatory gene expression and preventing microglial activation [40].
Lipopolysaccharides (LPS)	Gram-negative bacteria	Promotes neuroinflammation and BBB permeability, exacerbating neurodegeneration	LPS activates TLR4 receptors on microglia and astrocytes, leading to IL-6 and TNF-α production via NF-κB activation [48].
Tryptophan Metabolites (e.g., Indoles)	Tryptophan metabolism by gut bacteria	Modulates immune response; can exert neuroprotective effects	Indoles can inhibit NF-κB activation, reducing neuroinflammation and promoting neuronal survival [49].
Bile Acids	Microbial transformation of bile acids	Modulates microglial activation; impacts inflammation and neuroprotection	Bile acids activate G-protein-coupled receptors (GPCRs) such as TGR5, which can modulate immune cell signaling in the brain [50].
Amines (e.g., Histamine)	Bacterial decarboxylation of amino acids	Can induce neuroinflammation in excess; role in Parkinson’s disease (PD)	Histamine binds to H1 receptors on microglia, triggering pro-inflammatory cytokine release and potentially aggravating neurodegeneration [51].
Aryl Hydrocarbon Receptor Ligands	Metabolites of tryptophan and other aromatic compounds	Modulates immune response and neuroinflammation	These metabolites activate the aryl hydrocarbon receptor (AhR), which influences neuroimmune responses and can suppress inflammation in certain contexts [52].
GABA (Gamma-aminobutyric acid)	Gut microbiota producing GABA	Neuroprotective; regulates neuroinflammation and promotes neuronal survival	GABA modulates GABA receptors on neurons and glial cells, suppressing pro-inflammatory cytokine production and enhancing neuroprotection [53].
Polyamines (e.g., Spermidine)	Bacterial synthesis of polyamines	Protects against oxidative stress and neurodegeneration	Spermidine activates autophagy, reducing cellular stress and promoting neuronal health by mitigating protein aggregation and inflammation [54].
Catechols (e.g., Dopamine)	Gut microbiota influencing dopamine pathways	Potential influence on Parkinson’s disease progression and motor control	Gut-derived dopamine metabolites can impact the brain’s dopaminergic system, influencing neurodegenerative diseases such as Parkinson’s [55].
P-cresol	Bacterial metabolism of aromatic amino acids	Increases oxidative stress, exacerbating neurodegeneration	P-cresol can activate inflammatory pathways and generate reactive oxygen species (ROS), contributing to neuronal damage in neurodegenerative diseases [56].
Amino Acids (e.g., Glutamate)	Bacterial conversion of dietary proteins	Modulates neuronal excitotoxicity; potentially harmful in excess	Excess glutamate from gut microbial metabolism can increase neuronal excitotoxicity and promote neurodegenerative conditions like Alzheimer’s [57].
Polyphenols (e.g., Resveratrol)	Microbial fermentation of polyphenol-rich foods	Antioxidant and anti-inflammatory properties; neuroprotective effects	Resveratrol and other polyphenols can modulate gut microbiota composition and reduce neuroinflammation, promoting brain health and potentially slowing degeneration [58].
Vitamins (e.g., B12, D)	Gut bacteria and fermentation processes	Regulate immune response and neuronal function	Vitamin B12 is essential for nerve function, while Vitamin D modulates immune pathways that influence neuroinflammation and neurodegeneration [59].
Fatty Acids (Omega-3, Omega-6)	Fermentation of dietary fats	Modulate brain inflammation; protect against cognitive decline	Omega-3 fatty acids reduce the production of pro-inflammatory cytokines, whereas an imbalance (e.g., too many omega-6 fatty acids) can promote neuroinflammation [60].

**Table 2:** Dietary components and their effects on gut microbiota.

Dietary Component	Effect on Gut Microbiota
Polyphenols	Promote the growth of beneficial bacteria (e.g., *Bifidobacterium* sp., *Lactobacillus* sp.) and inhibit pathogenic bacteria [125].
Omega-3 Fatty Acids	Enhance gut microbiota diversity, promote anti-inflammatory bacteria, and reduce pro-inflammatory microbial species [126].
Fiber (Prebiotics)	Promote the growth of beneficial gut bacteria, such as *Bacteroides*, which ferment fiber into SCFAs [127].
Fermented Foods	Introduce probiotics, such as *Lactobacillus* sp. *Bifidobacterium* sp., to enhance gut microbiota balance and diversity [128].
Polyunsaturated Fatty Acids	Can alter gut microbiota composition, sometimes promoting the growth of beneficial microbes while reducing harmful bacteria [126].
High-Fat, Low-Fiber Diets	This can lead to dysbiosis, decreasing the diversity of beneficial bacteria and promoting the growth of harmful microbes like *Firmicutes* [129].
Antioxidants (e.g., Vitamin C, E)	Enhance the growth of beneficial bacteria and reduce gut inflammation, supporting a balanced microbiota [130].
Probiotics	Directly introduce beneficial bacteria that can improve gut health, enhance microbial diversity, and reduce inflammation [131].
High-Sugar Diets	An increased abundance of pathogenic bacteria, such as *Firmicutes*, and can lead to inflammation and gut dysbiosis [132].
High-Protein Diets	It can alter the gut microbiome by enhancing the prevalence of protein-fermenting microflora, potentially promoting dysbiosis [133].

**Table 3:** Clinical Trials Investigating Diet-Microbiota Interventions in Alzheimer’s.

Study	Intervention	Target Group	Outcome
Analytics Study (2024)	Mediterranean diet	Elderly individuals at risk of Alzheimer’s	Improved cognitive function and reduced neuroinflammation [153].
Omega-3 and Probiotic Trial (2021)	Omega-3 supplementation + probiotics	Alzheimer’s patients with mild cognitive impairment	Improvement in cognitive scores and gut microbiota diversity [154].
Prebiotic Intervention Trial (2021)	Prebiotics (inulin and fructooligosaccharides)	Elderly individuals with early-stage Alzheimer’s	Increased beneficial gut bacteria and reduced amyloid plaques [155].
Gut Microbiota Modulation Study (2025)	High-fiber diet and probiotic supplementation	Adults with early-stage Alzheimer’s disease	Significant improvement in cognitive function and gut health [156].
*Bifidobacterium* sp. and Cognitive Decline Study (2025)	*Bifidobacterium* sp. supplementation	Individuals with mild cognitive impairment	Increased gut microbial diversity and improved cognition [157].
Polyphenol-Rich Diet Trial (2025)	Polyphenol-rich foods (e.g., blueberries, green tea)	Adults at risk for Alzheimer’s	Enhancement in cognitive function and reduction of oxidative stress [158].
High-Protein Diet and Neurodegeneration Study (2020)	High-protein, low-carb diet	Alzheimer’s patients with advanced stages	Modulation of gut microbiota towards anti-inflammatory species [133].

**Table 4:** Potential probiotic and prebiotic strategies for cognitive health.

Strategy	Probiotic/ Prebiotic Type	Mechanism of Action	Potential Cognitive Benefit
*Lactobacillus* species	Probiotic	Modulates gut microbiota, reduces inflammation, increases SCFAs	Enhances memory, reduces neuroinflammation [198]
*Bifidobacterium* species	Probiotic	Supports gut barrier integrity, balances gut microbiota	Improves cognitive function, reduces amyloid plaque formation [199]
Inulin (prebiotic fiber)	Prebiotic	Stimulates growth of beneficial bacteria, enhances SCFA production	Reduces neuroinflammation, enhances cognitive performance [200]
Fructooligosaccharides (FOS)	Prebiotic	Promotes beneficial microbiota, increases SCFAs, reduces endotoxins	Improves synaptic plasticity, reduces cognitive decline [201]
*Lactobacillus rhamnosus* GR1	Probiotic	Reduces gut permeability, modulates immune response	Improves mood and cognitive functions [202]
Prebiotic dietary fiber (e.g., pectin)	Prebiotic	Stimulates beneficial bacteria, enhances microbial diversity	Enhances neuroprotection via gut–brain signaling [203]
*Bifidobacterium longum*	Probiotic	Modulates immune response, enhances short-chain fatty acid production	Reduces neuroinflammation, improves memory and cognitive function [204]
Galacto-oligosaccharides (GOS)	Prebiotic	Increases beneficial microbiota, enhances gut barrier function	Modulates gut–brain signaling, promotes neuroprotective effects [205]

**Table 5:** Current gaps in knowledge and future research directions.

Research Gap	Description	Future Research Directions
Longitudinal Microbiome Studies	Limited knowledge of how gut microbiota evolves in Alzheimer’s disease.	Large-scale, long-term microbiome studies should be conducted to identify causal relationships between microbiota shifts and disease development [222].
Microbiome–Drug Interactions	Insufficient knowledge on how the microbiome affects the efficacy of pharmaceutical treatments for Alzheimer’s.	Investigate the interactions between gut microbiota and Alzheimer’s drugs to optimize therapeutic strategies [223].
Microbial Biomarkers for Early Diagnosis	Lack of reliable biomarkers for early detection of Alzheimer’s based on microbiome composition.	Developing microbiome-based diagnostic tools for early detection and monitoring of Alzheimer’s progression [224].
Personalized Diet–Microbiome Interventions	Variability in individual responses to diet-based interventions.	Conduct precision nutrition studies to personalize dietary recommendations based on microbiome profiles for cognitive health [213].
Mechanisms Linking Gut Microbiota to Brain Pathology	Limited conception of the specific microbial metabolites and pathways involved in Alzheimer’s pathology.	Investigate the molecular mechanisms by which microbiome-derived metabolites influence brain inflammation and protein accumulation (e.g., amyloid-β, tau) [225].
Impact of Gut Dysbiosis on Blood–Brain Barrier (BBB)	Inadequate insight on how gut dysbiosis contributes to BBB disruption in Alzheimer’s.	Study the effects of microbiome modulation on BBB integrity and its role in neurodegenerative diseases [226].
Therapeutic Potential of Fecal Microbiota Transplantation (FMT)	Insufficient evidence on the effectiveness of FMT in treating Alzheimer’s.	Conduct clinical trials to evaluate the safety and efficacy of FMT as a therapy for Alzheimer’s [227].
Long-Term Effects of Probiotics and Prebiotics	Uncertainty about the long-term impact of probiotics and prebiotics on Alzheimer’s progression.	Long-term studies should assess probiotic and prebiotic interventions’ sustained benefits and risks in Alzheimer’s patients [156].
Microbiome-Based Personalized Medicine	Lack of comprehensive, personalized treatment options based on microbiome profiling.	Develop personalized treatment strategies using microbiome-based therapies tailored to individual profiles for neurodegenerative diseases [189].
Comprehensive Multi-Omics Approaches	Insufficient integration of multi-omics data to understand the full complexity of the gut–brain axis.	Integrate genomics, metabolomics, and proteomics data to understand better the gut–brain interactions and Alzheimer’s pathology [228].

## Data Availability

No new data were created or analyzed in this study.

## Abbreviations

The following abbreviations are used in this manuscript:

AD	Alzheimer’s disease
SCFAs	Short-chain fatty acids
BBB	Blood–brain barrier
FMT	Fecal microbiota transplantation
LPS	Lipopolysaccharides
NMDA	N-methyl-D-aspartate
FXR	Farnesoid X receptor
TGR5	G-protein receptor 5
CNS	Central nervous system
IL-6	Interleukin-6
TNF-α	Tumor necrosis factor-alpha
NF-κB	Nuclear factor kappa B
TLR4	Toll-like receptor 4
HDACs	Histone deacetylases
GPCRs	G-protein-coupled receptors
PD	Parkinson’s disease
AhR	Aryl hydrocarbon receptor
GABA	Gamma-aminobutyric acid
ROS	Reactive oxygen species
EPA	Eicosapentaenoic acid
DHA	Docosahexaenoic acid
Aβ	Amyloid-β
GSK-3β	Glycogen synthase kinase 3 beta
CDK5	Cyclin-dependent kinase 5
FOS	Fructooligosaccharides

## References

[1] Wang S., Jiang Y., Yang A., Meng F., Zhang J. (2024). The Expanding Burden of Neurodegenerative Diseases: An Unmet Medical and Social Need. Aging Dis..

[2] (2024). 2024 Alzheimer’s Disease Facts and Figures. Alzheimer’s Dement..

[3] Lastuka A., Bliss E., Breshock M.R., Iannucci V.C., Sogge W., Taylor K.V., Pedroza P., Dieleman J.L. (2024). Societal Costs of Dementia: 204 Countries, 2000–2019. J. Alzheimer’s Dis..

[4] (2020). Economic Burden of Alzheimer Disease and Managed Care Considerations. Am. J. Manag. Care.

[5] Lohiya A., Dhaniwala N., Dudhekar U., Goyal S., Patel S.K. (2023). A Comprehensive Review of Treatment Strategies for Early Avascular Necrosis. Cureus.

[6] Pucci C., Martinelli C., Ciofani G. (2019). Innovative Approaches for Cancer Treatment: Current Perspectives and New Challenges. Ecancermedicalscience.

[7] O’Riordan K.J., Moloney G.M., Keane L., Clarke G., Cryan J.F. (2025). The Gut Microbiota-Immune-Brain Axis: Therapeutic Implications. Cell Rep. Med..

[8] Mostafavi Abdolmaleky H., Zhou J.-R. (2024). Gut Microbiota Dysbiosis, Oxidative Stress, Inflammation, and Epigenetic Alterations in Metabolic Diseases. Antioxidants.

[9] Chandra S., Sisodia S.S., Vassar R.J. (2023). The Gut Microbiome in Alzheimer’s Disease: What We Know and What Remains to Be Explored. Mol. Neurodegener..

[10] Qian X., Xie R., Liu X., Chen S., Tang H. (2022). Mechanisms of Short-Chain Fatty Acids Derived from Gut Microbiota in Alzheimer’s Disease. Aging Dis..

[11] Ullah R., Park T.J., Huang X., Kim M.O. (2021). Abnormal Amyloid Beta Metabolism in Systemic Abnormalities and Alzheimer’s Pathology: Insights and Therapeutic Approaches from Periphery. Ageing Res. Rev..

[12] Yang J., Liang J., Hu N., He N., Liu B., Liu G., Qin Y. (2024). The Gut Microbiota Modulates Neuroinflammation in Alzheimer’s Disease: Elucidating Crucial Factors and Mechanistic Underpinnings. CNS Neurosci. Ther..

[13] Hou K., Wu Z.-X., Chen X.-Y., Wang J.-Q., Zhang D., Xiao C., Zhu D., Koya J.B., Wei L., Li J. (2022). Microbiota in Health and Diseases. Signal Transduct. Target. Ther..

[14] Mateo D., Carrión N., Cabrera C., Heredia L., Marquès M., Forcadell-Ferreres E., Pino M., Zaragoza J., Moral A., Cavallé L. (2024). Gut Microbiota Alterations in Alzheimer’s Disease: Relation with Cognitive Impairment and Mediterranean Lifestyle. Microorganisms.

[15] Khatoon S., Kalam N., Rashid S., Bano G. (2023). Effects of Gut Microbiota on Neurodegenerative Diseases. Front. Aging Neurosci..

[16] Fusco W., Lorenzo M.B., Cintoni M., Porcari S., Rinninella E., Kaitsas F., Lener E., Mele M.C., Gasbarrini A., Collado M.C. (2023). Short-Chain Fatty-Acid-Producing Bacteria: Key Components of the Human Gut Microbiota. Nutrients.

[17] O’Riordan K.J., Collins M.K., Moloney G.M., Knox E.G., Aburto M.R., Fülling C., Morley S.J., Clarke G., Schellekens H., Cryan J.F. (2022). Short Chain Fatty Acids: Microbial Metabolites for Gut-Brain Axis Signalling. Mol. Cell. Endocrinol..

[18] Koutsokostas C., Merkouris E., Goulas A., Aidinopoulou K., Sini N., Dimaras T., Tsiptsios D., Mueller C., Nystazaki M., Tsamakis K. (2024). Gut Microbes Associated with Neurodegenerative Disorders: A Comprehensive Review of the Literature. Microorganisms.

[19] Zhang Y., Geng R., Tu Q. (2021). Gut Microbial Involvement in Alzheimer’s Disease Pathogenesis. Aging.

[20] Missiego-Beltrán J., Beltrán-Velasco A.I. (2024). The Role of Microbial Metabolites in the Progression of Neurodegenerative Diseases—Therapeutic Approaches: A Comprehensive Review. Int. J. Mol. Sci..

[21] Swer N.M., Venkidesh B.S., Murali T.S., Mumbrekar K.D. (2023). Gut Microbiota-Derived Metabolites and Their Importance in Neurological Disorders. Mol. Biol. Rep..

[22] Xiong R.-G., Zhou D.-D., Wu S.-X., Huang S.-Y., Saimaiti A., Yang Z.-J., Shang A., Zhao C.-N., Gan R.-Y., Li H.-B. (2022). Health Benefits and Side Effects of Short-Chain Fatty Acids. Foods.

[23] Singh V., Lee G., Son H., Koh H., Kim E.S., Unno T., Shin J.-H. (2023). Butyrate Producers, “The Sentinel of Gut”: Their Intestinal Significance with and beyond Butyrate, and Prospective Use as Microbial Therapeutics. Front. Microbiol..

[24] Zhou C., Zhao D., Wu C., Wu Z., Zhang W., Chen S., Zhao X., Wu S. (2024). Role of Histone Deacetylase Inhibitors in Non-Neoplastic Diseases. Heliyon.

[25] Fock E., Parnova R. (2023). Mechanisms of Blood–Brain Barrier Protection by Microbiota-Derived Short-Chain Fatty Acids. Cells.

[26] Chen G., Shi F., Yin W., Guo Y., Liu A., Shuai J., Sun J. (2022). Gut Microbiota Dysbiosis: The Potential Mechanisms by Which Alcohol Disrupts Gut and Brain Functions. Front. Microbiol..

[27] Mossad O., Erny D. (2020). The Microbiota–Microglia Axis in Central Nervous System Disorders. Brain Pathol..

[28] Wu S., Liu X., Jiang R., Yan X., Ling Z. (2021). Roles and Mechanisms of Gut Microbiota in Patients with Alzheimer’s Disease. Front. Aging Neurosci..

[29] Shaw C., Hess M., Weimer B.C. (2023). Microbial-Derived Tryptophan Metabolites and Their Role in Neurological Disease: Anthranilic Acid and Anthranilic Acid Derivatives. Microorganisms.

[30] Meier T.B., Savitz J. (2022). The Kynurenine Pathway in Traumatic Brain Injury: Implications for Psychiatric Outcomes. Biol. Psychiatry.

[31] Uceda S., Echeverry-Alzate V., Reiriz-Rojas M., Martínez-Miguel E., Pérez-Curiel A., Gómez-Senent S., Beltrán-Velasco A.I. (2023). Gut Microbial Metabolome and Dysbiosis in Neurodegenerative Diseases: Psychobiotics and Fecal Microbiota Transplantation as a Therapeutic Approach—A Comprehensive Narrative Review. Int. J. Mol. Sci..

[32] Akram N., Faisal Z., Irfan R., Shah Y.A., Batool S.A., Zahid T., Zulfiqar A., Fatima A., Jahan Q., Tariq H. (2024). Exploring the serotonin-probiotics-gut Health Axis: A Review of Current Evidence and Potential Mechanisms. Food Sci. Nutr..

[33] Gao K., Mu C., Farzi A., Zhu W. (2020). Tryptophan Metabolism: A Link Between the Gut Microbiota and Brain. Adv. Nutr..

[34] Kciuk M., Kruczkowska W., Gałęziewska J., Wanke K., Kałuzińska-Kołat Ż., Aleksandrowicz M., Kontek R. (2024). Alzheimer’s Disease as Type 3 Diabetes: Understanding the Link and Implications. Int. J. Mol. Sci..

[35] Tanaka M., Szabó Á., Vécsei L. (2024). Redefining Roles: A Paradigm Shift in Tryptophan–Kynurenine Metabolism for Innovative Clinical Applications. Int. J. Mol. Sci..

[36] Liang Y., Xie S., He Y., Xu M., Qiao X., Zhu Y., Wu W. (2022). Kynurenine Pathway Metabolites as Biomarkers in Alzheimer’s Disease. Dis. Mark..

[37] Sabahat S.E., Saqib M., Talib M., Shaikh T.G., Khan T., Kailash S.J. (2024). Bile Acid Modulation by Gut Microbiota: A Bridge to Understanding Cognitive Health. Ann. Med. Surg..

[38] Grant S.M., DeMorrow S. (2020). Bile Acid Signaling in Neurodegenerative and Neurological Disorders. Int. J. Mol. Sci..

[39] Jia M., Fan Y., Ma Q., Yang D., Wang Y., He X., Zhao B., Zhan X., Qi Z., Ren Y. (2024). Gut Microbiota Dysbiosis Promotes Cognitive Impairment via Bile Acid Metabolism in Major Depressive Disorder. Transl. Psychiatry.

[40] Silva Y.P., Bernardi A., Frozza R.L. (2020). The Role of Short-Chain Fatty Acids From Gut Microbiota in Gut-Brain Communication. Front. Endocrinol..

[41] Suleiman Khoury Z., Sohail F., Wang J., Mendoza M., Raake M., Tahoor Silat M., Reddy Bathinapatta M., Sadeghzadegan A., Meghana P., Paul J. (2024). Neuroinflammation: A Critical Factor in Neurodegenerative Disorders. Cureus.

[42] Shahini A., Shahini A. (2023). Role of Interleukin-6-Mediated Inflammation in the Pathogenesis of Inflammatory Bowel Disease: Focus on the Available Therapeutic Approaches and Gut Microbiome. J. Cell Commun. Signal..

[43] Bairamian D., Sha S., Rolhion N., Sokol H., Dorothée G., Lemere C.A., Krantic S. (2022). Microbiota in Neuroinflammation and Synaptic Dysfunction: A Focus on Alzheimer’s Disease. Mol. Neurodegener..

[44] Anand N., Gorantla V.R., Chidambaram S.B. (2022). The Role of Gut Dysbiosis in the Pathophysiology of Neuropsychiatric Disorders. Cells.

[45] Guo Q., Jin Y., Chen X., Ye X., Shen X., Lin M., Zeng C., Zhou T., Zhang J. (2024). NF-ΚB in Biology and Targeted Therapy: New Insights and Translational Implications. Signal Transduct. Target. Ther..

[46] Xie L., Wu Q., Li K., Khan M.A.S., Zhang A., Sinha B., Li S., Chang S.L., Brody D.L., Grinstaff M.W. (2024). Tryptophan Metabolism in Alzheimer’s Disease with the Involvement of Microglia and Astrocyte Crosstalk and Gut-Brain Axis. Aging Dis..

[47] Park K.J., Gao Y. (2024). Gut-Brain Axis and Neurodegeneration: Mechanisms and Therapeutic Potentials. Front. Neurosci..

[48] Peng X., Luo Z., He S., Zhang L., Li Y. (2021). Blood-Brain Barrier Disruption by Lipopolysaccharide and Sepsis-Associated Encephalopathy. Front. Cell. Infect. Microbiol..

[49] Li S. (2023). Modulation of Immunity by Tryptophan Microbial Metabolites. Front. Nutr..

[50] Keitel V., Stindt J., Häussinger D. (2019). Bile Acid-Activated Receptors: GPBAR1 (TGR5) and Other G Protein-Coupled Receptors.

[51] Comas-Basté O., Luz Latorre-Moratalla M., Sánchez-Pérez S., Teresa Veciana-Nogués M., del Carmen Vidal-Carou M. (2019). Histamine and Other Biogenic Amines in Food. From Scombroid Poisoning to Histamine Intolerance. Biogenic Amines.

[52] Anderson G., Carbone A., Mazzoccoli G. (2021). Tryptophan Metabolites and Aryl Hydrocarbon Receptor in Severe Acute Respiratory Syndrome, Coronavirus-2 (SARS-CoV-2) Pathophysiology. Int. J. Mol. Sci..

[53] Fashogbon R.O., Samson O.J., Awotundun T.A., Olanbiwoninu A.A., Adebayo-Tayo B.C. (2024). Microbial Gamma-Aminobutyric Acid Synthesis: A Promising Approach for Functional Food and Pharmaceutical Applications. Lett. Appl. Microbiol..

[54] Smirnova O.A., Isaguliants M.G., Hyvonen M.T., Keinanen T.A., Tunitskaya V.L., Vepsalainen J., Alhonen L., Kochetkov S.N., Ivanov A.V. (2012). Chemically Induced Oxidative Stress Increases Polyamine Levels by Activating the Transcription of Ornithine Decarboxylase and Spermidine/Spermine-N1-Acetyltransferase in Human Hepatoma HUH7 Cells. Biochimie.

[55] Hamamah S., Aghazarian A., Nazaryan A., Hajnal A., Covasa M. (2022). Role of Microbiota-Gut-Brain Axis in Regulating Dopaminergic Signaling. Biomedicines.

[56] Singh A., Kukreti R., Saso L., Kukreti S. (2019). Oxidative Stress: A Key Modulator in Neurodegenerative Diseases. Molecules.

[57] Gruenbaum B.F., Merchant K.S., Zlotnik A., Boyko M. (2024). Gut Microbiome Modulation of Glutamate Dynamics: Implications for Brain Health and Neurotoxicity. Nutrients.

[58] Ciupei D., Colişar A., Leopold L., Stănilă A., Diaconeasa Z.M. (2024). Polyphenols: From Classification to Therapeutic Potential and Bioavailability. Foods.

[59] Sarb O.-F., Sarb A.-D., Iacobescu M., Vlad I.-M., Milaciu M.-V., Ciurmarnean L., Vacaras V., Tantau A.-I. (2024). From Gut to Brain: Uncovering Potential Serum Biomarkers Connecting Inflammatory Bowel Diseases to Neurodegenerative Diseases. Int. J. Mol. Sci..

[60] Innes J.K., Calder P.C. (2018). Omega-6 Fatty Acids and Inflammation. Prostaglandins Leukot. Essent. Fat. Acids.

[61] Zhang S., Gan L., Cao F., Wang H., Gong P., Ma C., Ren L., Lin Y., Lin X. (2022). The Barrier and Interface Mechanisms of the Brain Barrier, and Brain Drug Delivery. Brain Res. Bull..

[62] Duan H., Wang L., Huangfu M., Li H. (2023). The Impact of Microbiota-Derived Short-Chain Fatty Acids on Macrophage Activities in Disease: Mechanisms and Therapeutic Potentials. Biomed. Pharmacother..

[63] Liu M., Peng R., Tian C., Shi J., Ma J., Shi R., Qi X., Zhao R., Guan H. (2024). Effects of the Gut Microbiota and Its Metabolite Short-Chain Fatty Acids on Endometriosis. Front. Cell. Infect. Microbiol..

[64] Chandrasekaran P., Weiskirchen S., Weiskirchen R. (2024). Effects of Probiotics on Gut Microbiota: An Overview. Int. J. Mol. Sci..

[65] Beltran-Velasco A.I., Clemente-Suárez V.J. (2025). Impact of Peripheral Inflammation on Blood–Brain Barrier Dysfunction and Its Role in Neurodegenerative Diseases. Int. J. Mol. Sci..

[66] Ashique S., Mohanto S., Ahmed M.G., Mishra N., Garg A., Chellappan D.K., Omara T., Iqbal S., Kahwa I. (2024). Gut-Brain Axis: A Cutting-Edge Approach to Target Neurological Disorders and Potential Synbiotic Application. Heliyon.

[67] Zhao Y., Gan L., Ren L., Lin Y., Ma C., Lin X. (2022). Factors Influencing the Blood-Brain Barrier Permeability. Brain Res..

[68] Mou Y., Du Y., Zhou L., Yue J., Hu X., Liu Y., Chen S., Lin X., Zhang G., Xiao H. (2022). Gut Microbiota Interact with the Brain Through Systemic Chronic Inflammation: Implications on Neuroinflammation, Neurodegeneration, and Aging. Front. Immunol..

[69] Kim M.E., Lee J.S. (2024). Mechanisms and Emerging Regulators of Neuroinflammation: Exploring New Therapeutic Strategies for Neurological Disorders. Curr. Issues Mol. Biol..

[70] Chen Y., He Y., Han J., Wei W., Chen F. (2023). Blood-Brain Barrier Dysfunction and Alzheimer’s Disease: Associations, Pathogenic Mechanisms, and Therapeutic Potential. Front. Aging Neurosci..

[71] Rob M., Yousef M., Lakshmanan A.P., Mahboob A., Terranegra A., Chaari A. (2025). Microbial Signatures and Therapeutic Strategies in Neurodegenerative Diseases. Biomed. Pharmacother..

[72] Kustrimovic N., Balkhi S., Bilato G., Mortara L. (2024). Gut Microbiota and Immune System Dynamics in Parkinson’s and Alzheimer’s Diseases. Int. J. Mol. Sci..

[73] Yoo J., Groer M., Dutra S., Sarkar A., McSkimming D. (2020). Gut Microbiota and Immune System Interactions. Microorganisms.

[74] Adamu A., Li S., Gao F., Xue G. (2024). The Role of Neuroinflammation in Neurodegenerative Diseases: Current Understanding and Future Therapeutic Targets. Front. Aging Neurosci..

[75] Xu G., Dong F., Su L., Tan Z.-X., Lei M., Li L., Wen D., Zhang F. (2024). The Role and Therapeutic Potential of Nuclear Factor ΚB (NF-ΚB) in Ischemic Stroke. Biomed. Pharmacother..

[76] Anilkumar S., Wright-Jin E. (2024). NF-ΚB as an Inducible Regulator of Inflammation in the Central Nervous System. Cells.

[77] Popescu C., Munteanu C., Anghelescu A., Ciobanu V., Spînu A., Andone I., Mandu M., Bistriceanu R., Băilă M., Postoiu R.-L. (2024). Novelties on Neuroinflammation in Alzheimer’s Disease–Focus on Gut and Oral Microbiota Involvement. Int. J. Mol. Sci..

[78] Violi F., Cammisotto V., Bartimoccia S., Pignatelli P., Carnevale R., Nocella C. (2023). Gut-Derived Low-Grade Endotoxaemia, Atherothrombosis and Cardiovascular Disease. Nat. Rev. Cardiol..

[79] Kim S., Jung U.J., Kim S.R. (2025). The Crucial Role of the Blood–Brain Barrier in Neurodegenerative Diseases: Mechanisms of Disruption and Therapeutic Implications. J. Clin. Med..

[80] Chen T., Dai Y., Hu C., Lin Z., Wang S., Yang J., Zeng L., Li S., Li W. (2024). Cellular and Molecular Mechanisms of the Blood–Brain Barrier Dysfunction in Neurodegenerative Diseases. Fluids Barriers CNS.

[81] Che Mohd Nassir C.M.N., Che Ramli M.D., Mohamad Ghazali M., Jaffer U., Abdul Hamid H., Mehat M.Z., Hein Z.M. (2024). The Microbiota–Gut–Brain Axis: Key Mechanisms Driving Glymphopathy and Cerebral Small Vessel Disease. Life.

[82] Clerici L., Bottari D., Bottari B. (2025). Gut Microbiome, Diet and Depression: Literature Review of Microbiological, Nutritional and Neuroscientific Aspects. Curr. Nutr. Rep..

[83] Clemente-Suárez V.J., Beltrán-Velasco A.I., Redondo-Flórez L., Martín-Rodríguez A., Tornero-Aguilera J.F. (2023). Global Impacts of Western Diet and Its Effects on Metabolism and Health: A Narrative Review. Nutrients.

[84] Severino A., Tohumcu E., Tamai L., Dargenio P., Porcari S., Rondinella D., Venturini I., Maida M., Gasbarrini A., Cammarota G. (2024). The Microbiome-Driven Impact of Western Diet in the Development of Noncommunicable Chronic Disorders. Best Pract. Res. Clin. Gastroenterol..

[85] Song M., Bai Y., Song F. (2025). High-Fat Diet and Neuroinflammation: The Role of Mitochondria. Pharmacol. Res..

[86] Edo G.I., Nwachukwu S.C., Akpoghelie P.O., Mafe A.N., Isoje E.F., Igbuku U.A., Yousif E., Zainulabdeen K., Jikah A.N., Owheruo J.O. An Overview of the Nutritional Composition, Bioactivities and Applications of Chinese Yam (Dioscoreas Oppositae). Ecol. Front..

[87] Mafe A.N., Edo G.I., Akpoghelie P.O., Yousif E., Gaaz T.S., Opiti R.A., Onyibe P.N., Owheruo J.O., Isoje E.F., Igbuku U.A. (2024). Pepper Soup: A Cultural and Culinary Exploration of a Traditional Nigerian Dish, with a Focus on Health Benefits and Antimicrobial Activity. Int. J. Gastron. Food Sci..

[88] Dominguez L.J., Veronese N., Di Bella G., Cusumano C., Parisi A., Tagliaferri F., Ciriminna S., Barbagallo M. (2023). Mediterranean Diet in the Management and Prevention of Obesity. Exp. Gerontol..

[89] Randeni N., Bordiga M., Xu B. (2024). A Comprehensive Review of the Triangular Relationship among Diet–Gut Microbiota–Inflammation. Int. J. Mol. Sci..

[90] Picone P., Girgenti A., Buttacavoli M., Nuzzo D. (2024). Enriching the Mediterranean Diet Could Nourish the Brain More Effectively. Front. Nutr..

[91] Zhou J., Tang M., Li W., Fang R., Tang C., Wang Q. (2024). Diet and Physical Activity Influence the Composition of Gut Microbiota, Benefit on Alzheimer’s Disease. Food Sci. Hum. Wellness.

[92] Bailén M., Bressa C., Martínez-López S., González-Soltero R., Montalvo Lominchar M.G., San Juan C., Larrosa M. (2020). Microbiota Features Associated with a High-Fat/Low-Fiber Diet in Healthy Adults. Front. Nutr..

[93] Kang G.G., Trevaskis N.L., Murphy A.J., Febbraio M.A. (2023). Diet-Induced Gut Dysbiosis and Inflammation: Key Drivers of Obesity-Driven NASH. iScience.

[94] Lotz S.K., Blackhurst B.M., Reagin K.L., Funk K.E. (2021). Microbial Infections Are a Risk Factor for Neurodegenerative Diseases. Front. Cell. Neurosci..

[95] Młynarska E., Jakubowska P., Frąk W., Gajewska A., Sornowska J., Skwira S., Wasiak J., Rysz J., Franczyk B. (2024). Associations of Microbiota and Nutrition with Cognitive Impairment in Diseases. Nutrients.

[96] Park G., Kadyan S., Hochuli N., Pollak J., Wang B., Salazar G., Chakrabarty P., Efron P., Sheffler J., Nagpal R. (2024). A Modified Mediterranean-Style Diet Enhances Brain Function via Specific Gut-Microbiome-Brain Mechanisms. Gut Microbes.

[97] Hyży A., Rozenek H., Gondek E., Jaworski M. (2025). Effect of Antioxidants on the Gut Microbiome Profile and Brain Functions: A Review of Randomized Controlled Trial Studies. Foods.

[98] Mafe A.N., Iruoghene Edo G., Akpoghelie P.O., Gaaz T.S., Yousif E., Zainulabdeen K., Isoje E.F., Igbuku U.A., Opiti R.A., Garba Y. (2025). Probiotics and Food Bioactives: Unraveling Their Impact on Gut Microbiome, Inflammation, and Metabolic Health. Probiotics Antimicrob. Proteins.

[99] Mursal M., Kumar A., Hasan S.M., Hussain S., Singh K., Kushwaha S.P., Arif M., Kumar Singh R., Singh D., Mohammad A. (2024). Role of Natural Bioactive Compounds in the Management of Neurodegenerative Disorders. Intell. Pharm..

[100] Magrone T., Magrone M., Russo M.A., Jirillo E. (2019). Recent Advances on the Anti-Inflammatory and Antioxidant Properties of Red Grape Polyphenols: In Vitro and In Vivo Studies. Antioxidants.

[101] Thapa R., Moglad E., Afzal M., Gupta G., Bhat A.A., Hassan almalki W., Kazmi I., Alzarea S.I., Pant K., Singh T.G. (2024). The Role of Sirtuin 1 in Ageing and Neurodegenerative Disease: A Molecular Perspective. Ageing Res. Rev..

[102] Lagoa R., Rajan L., Violante C., Babiaka S.B., Marques-da-Silva D., Kapoor B., Reis F., Atanasov A.G. (2025). Application of Curcuminoids in Inflammatory, Neurodegenerative and Aging Conditions—Pharmacological Potential and Bioengineering Approaches to Improve Efficiency. Biotechnol. Adv..

[103] Chaudhary P., Janmeda P., Docea A.O., Yeskaliyeva B., Abdull Razis A.F., Modu B., Calina D., Sharifi-Rad J. (2023). Oxidative Stress, Free Radicals and Antioxidants: Potential Crosstalk in the Pathophysiology of Human Diseases. Front. Chem..

[104] Zinkow A., Grodzicki W., Czerwińska M., Dziendzikowska K. (2024). Molecular Mechanisms Linking Omega-3 Fatty Acids and the Gut–Brain Axis. Molecules.

[105] Banaszak M., Dobrzyńska M., Kawka A., Górna I., Woźniak D., Przysławski J., Drzymała-Czyż S. (2024). Role of Omega-3 Fatty Acids Eicosapentaenoic (EPA) and Docosahexaenoic (DHA) as Modulatory and Anti-Inflammatory Agents in Noncommunicable Diet-Related Diseases—Reports from the Last 10 Years. Clin. Nutr. ESPEN.

[106] Kumar V., Rohilla A., Ahire J.J. (2025). Omega-3 Fatty Acids and the Gut Microbiome: A New Frontier in Cardiovascular Disease Prevention. Discov. Med..

[107] Berding K., Vlckova K., Marx W., Schellekens H., Stanton C., Clarke G., Jacka F., Dinan T.G., Cryan J.F. (2021). Diet and the Microbiota–Gut–Brain Axis: Sowing the Seeds of Good Mental Health. Adv. Nutr..

[108] Singh A., Yau Y.F., Leung K.S., El-Nezami H., Lee J.C.-Y. (2020). Interaction of Polyphenols as Antioxidant and Anti-Inflammatory Compounds in Brain–Liver–Gut Axis. Antioxidants.

[109] Mafe A.N., Edo G.I., Majeed O.S., Gaaz T.S., Akpoghelie P.O., Isoje E.F., Igbuku U.A., Owheruo J.O., Opiti R.A., Garba Y. (2025). A Review on Probiotics and Dietary Bioactives: Insights on Metabolic Well-Being, Gut Microbiota, and Inflammatory Responses. Food Chem. Adv..

[110] Winiarska-Mieczan A., Kwiecień M., Jachimowicz-Rogowska K., Donaldson J., Tomaszewska E., Baranowska-Wójcik E. (2023). Anti-Inflammatory, Antioxidant, and Neuroprotective Effects of Polyphenols—Polyphenols as an Element of Diet Therapy in Depressive Disorders. Int. J. Mol. Sci..

[111] Dissanayaka D.M.S., Jayasena V., Rainey-Smith S.R., Martins R.N., Fernando W.M.A.D.B. (2024). The Role of Diet and Gut Microbiota in Alzheimer’s Disease. Nutrients.

[112] Siervo M., Shannon O.M., Llewellyn D.J., Stephan B.C., Fontana L. (2021). Mediterranean Diet and Cognitive Function: From Methodology to Mechanisms of Action. Free Radic. Biol. Med..

[113] Soldán M., Argalášová Ľ., Hadvinová L., Galileo B., Babjaková J. (2024). The Effect of Dietary Types on Gut Microbiota Composition and Development of Non-Communicable Diseases: A Narrative Review. Nutrients.

[114] Yavari M., Kalupahana N.S., Harris B.N., Ramalingam L., Zu Y., Kahathuduwa C.N., Moustaid-Moussa N. (2025). Mechanisms Linking Obesity, Insulin Resistance, and Alzheimer’s Disease: Effects of Polyphenols and Omega-3 Polyunsaturated Fatty Acids. Nutrients.

[115] Jimenez-García A.M., Villarino M., Arias N. (2024). A Systematic Review and Meta-analysis of Basal Microbiota and Cognitive Function in Alzheimer’s Disease: A Potential Target for Treatment or a Contributor to Disease Progression?. Alzheimer’s Dement. Diagn. Assess. Dis. Monit..

[116] Kumar S., Malviya R., Sundram S. (2024). Nutritional Neurology: Unraveling Cellular Mechanisms of Natural Supplements in Brain Health. Hum. Nutr. Metab..

[117] Stein R.A., Riber L. (2023). Epigenetic Effects of Short-Chain Fatty Acids from the Large Intestine on Host Cells. microLife.

[118] Prasanth M.I., Sivamaruthi B.S., Cheong C.S.Y., Verma K., Tencomnao T., Brimson J.M., Prasansuklab A. (2024). Role of Epigenetic Modulation in Neurodegenerative Diseases: Implications of Phytochemical Interventions. Antioxidants.

[119] Chandrasekaran V., Hediyal T.A., Anand N., Kendaganna P.H., Gorantla V.R., Mahalakshmi A.M., Ghanekar R.K., Yang J., Sakharkar M.K., Chidambaram S.B. (2023). Polyphenols, Autophagy and Neurodegenerative Diseases: A Review. Biomolecules.

[120] Gómez-Virgilio L., Silva-Lucero M.-C., Flores-Morelos D.-S., Gallardo-Nieto J., Lopez-Toledo G., Abarca-Fernandez A.-M., Zacapala-Gómez A.-E., Luna-Muñoz J., Montiel-Sosa F., Soto-Rojas L.O. (2022). Autophagy: A Key Regulator of Homeostasis and Disease: An Overview of Molecular Mechanisms and Modulators. Cells.

[121] Lin X., Liu W., Hu X., Liu Z., Wang F., Wang J. (2024). The Role of Polyphenols in Modulating Mitophagy: Implications for Therapeutic Interventions. Pharmacol. Res..

[122] Chen Y., Chen J., Xing Z., Peng C., Li D. (2024). Autophagy in Neuroinflammation: A Focus on Epigenetic Regulation. Aging Dis..

[123] Caetano-Silva M.E., Rund L., Hutchinson N.T., Woods J.A., Steelman A.J., Johnson R.W. (2023). Inhibition of Inflammatory Microglia by Dietary Fiber and Short-Chain Fatty Acids. Sci. Rep..

[124] Yan L., Guo M.-S., Zhang Y., Yu L., Wu J.-M., Tang Y., Ai W., Zhu F.-D., Law B.Y.-K., Chen Q. (2022). Dietary Plant Polyphenols as the Potential Drugs in Neurodegenerative Diseases: Current Evidence, Advances, and Opportunities. Oxid. Med. Cell. Longev..

[125] Singh A., Kaur P., Kumar M., Shafi S., Upadhyay P.K., Tiwari A., Tiwari V., Rangra N.K., Thirunavukkarasu V., Kumari S. (2025). The Role of Phytochemicals in Modulating the Gut Microbiota: Implications for Health and Disease. Med. Microecol..

[126] Fu Y., Wang Y., Gao H., Li D., Jiang R., Ge L., Tong C., Xu K. (2021). Associations among Dietary Omega-3 Polyunsaturated Fatty Acids, the Gut Microbiota, and Intestinal Immunity. Mediators Inflamm..

[127] Markowiak-Kopeć P., Śliżewska K. (2020). The Effect of Probiotics on the Production of Short-Chain Fatty Acids by Human Intestinal Microbiome. Nutrients.

[128] Vinderola G., Cotter P.D., Freitas M., Gueimonde M., Holscher H.D., Ruas-Madiedo P., Salminen S., Swanson K.S., Sanders M.E., Cifelli C.J. (2023). Fermented Foods: A Perspective on Their Role in Delivering Biotics. Front. Microbiol..

[129] Acevedo-Román A., Pagán-Zayas N., Velázquez-Rivera L.I., Torres-Ventura A.C., Godoy-Vitorino F. (2024). Insights into Gut Dysbiosis: Inflammatory Diseases, Obesity, and Restoration Approaches. Int. J. Mol. Sci..

[130] Naliyadhara N., Kumar A., Kumar Gangwar S., Nair Devanarayanan T., Hegde M., Alqahtani M.S., Abbas M., Sethi G., Kunnumakkara A. (2023). Interplay of Dietary Antioxidants and Gut Microbiome in Human Health: What Has Been Learnt Thus Far?. J. Funct. Foods.

[131] Bermúdez-Humarán L.G., Chassaing B., Langella P. (2024). Exploring the Interaction and Impact of Probiotic and Commensal Bacteria on Vitamins, Minerals and Short Chain Fatty Acids Metabolism. Microb. Cell Fact..

[132] Satokari R. (2020). High Intake of Sugar and the Balance between Pro- and Anti-Inflammatory Gut Bacteria. Nutrients.

[133] Wu S., Bhat Z., Gounder R., Mohamed Ahmed I., Al-Juhaimi F., Ding Y., Bekhit A. (2022). Effect of Dietary Protein and Processing on Gut Microbiota—A Systematic Review. Nutrients.

[134] Liu S., Gao J., Zhu M., Liu K., Zhang H.-L. (2020). Gut Microbiota and Dysbiosis in Alzheimer’s Disease: Implications for Pathogenesis and Treatment. Mol. Neurobiol..

[135] Frausto D.M., Forsyth C.B., Keshavarzian A., Voigt R.M. (2021). Dietary Regulation of Gut-Brain Axis in Alzheimer’s Disease: Importance of Microbiota Metabolites. Front. Neurosci..

[136] Kim H.S., Kim S., Shin S.J., Park Y.H., Nam Y., Kim C., Lee K., Kim S.-M., Jung I.D., Yang H.D. (2021). Gram-Negative Bacteria and Their Lipopolysaccharides in Alzheimer’s Disease: Pathologic Roles and Therapeutic Implications. Transl. Neurodegener..

[137] Ma B., Barathan M., Ng M.H., Law J.X. (2025). Oxidative Stress, Gut Microbiota, and Extracellular Vesicles: Interconnected Pathways and Therapeutic Potentials. Int. J. Mol. Sci..

[138] Sowmiya S., Dhivya L.S., Praveen R., Harikrishnan N., Singh A. (2024). Exploring the Potential of Probiotics in Alzheimer’s Disease and Gut Dysbiosis. IBRO Neurosci. Rep..

[139] Dhami M., Raj K., Singh S. (2023). Relevance of Gut Microbiota to Alzheimer’s Disease (AD): Potential Effects of Probiotic in Management of AD. Aging Health Res..

[140] Chen Y., Xu J., Chen Y. (2021). Regulation of Neurotransmitters by the Gut Microbiota and Effects on Cognition in Neurological Disorders. Nutrients.

[141] Nunzi E., Pariano M., Costantini C., Garaci E., Puccetti P., Romani L. (2025). Host–Microbe Serotonin Metabolism. Trends Endocrinol. Metab..

[142] Qu S., Yu Z., Zhou Y., Wang S., Jia M., Chen T., Zhang X. (2024). Gut Microbiota Modulates Neurotransmitter and Gut-Brain Signaling. Microbiol. Res..

[143] Salim S., Ahmad F., Banu A., Mohammad F. (2023). Gut Microbiome and Parkinson’s Disease: Perspective on Pathogenesis and Treatment. J. Adv. Res..

[144] Pluta R., Januszewski S. (2022). Gut Microbiota Neurotransmitters: Influence on Risk and Outcome of Ischemic Stroke. Neural Regen. Res..

[145] Rogers G.B., Keating D.J., Young R.L., Wong M.-L., Licinio J., Wesselingh S. (2016). From Gut Dysbiosis to Altered Brain Function and Mental Illness: Mechanisms and Pathways. Mol. Psychiatry.

[146] Wei W., Wang S., Xu C., Zhou X., Lian X., He L., Li K. (2022). Gut Microbiota, Pathogenic Proteins and Neurodegenerative Diseases. Front. Microbiol..

[147] Ajmal M.R. (2023). Protein Misfolding and Aggregation in Proteinopathies: Causes, Mechanism and Cellular Response. Diseases.

[148] Kim Y., Kim E.-K., Chey Y., Song M.-J., Jang H.H. (2023). Targeted Protein Degradation: Principles and Applications of the Proteasome. Cells.

[149] Zhao J., Duan L., Li J., Yao C., Wang G., Mi J., Yu Y., Ding L., Zhao Y., Yan G. (2024). New Insights into the Interplay between Autophagy, Gut Microbiota and Insulin Resistance in Metabolic Syndrome. Biomed. Pharmacother..

[150] Mitra S., Munni Y.A., Dash R., Sadhu T., Barua L., Islam M.A., Chowdhury D., Bhattacharjee D., Mazumder K., Moon I.S. (2023). Gut Microbiota in Autophagy Regulation: New Therapeutic Perspective in Neurodegeneration. Life.

[151] Solanki R., Karande A., Ranganathan P. (2023). Emerging Role of Gut Microbiota Dysbiosis in Neuroinflammation and Neurodegeneration. Front. Neurol..

[152] Jain A., Madkan S., Patil P. (2023). The Role of Gut Microbiota in Neurodegenerative Diseases: Current Insights and Therapeutic Implications. Cureus.

[153] Vaziri Y. (2024). The Mediterranean Diet: A Powerful Defense against Alzheimer Disease—A Comprehensive Review. Clin. Nutr. ESPEN.

[154] Białecka-Dębek A., Granda D., Szmidt M.K., Zielińska D. (2021). Gut Microbiota, Probiotic Interventions, and Cognitive Function in the Elderly: A Review of Current Knowledge. Nutrients.

[155] Kang J.W., Zivkovic A.M. (2021). The Potential Utility of Prebiotics to Modulate Alzheimer’s Disease: A Review of the Evidence. Microorganisms.

[156] Quansah M., David M.A., Martins R., El-Omar E., Aliberti S.M., Capunzo M., Jensen S.O., Tayebi M. (2025). The Beneficial Effects of Lactobacillus Strains on Gut Microbiome in Alzheimer’s Disease: A Systematic Review. Healthcare.

[157] Reiriz M., Beltrán-Velasco A.I., Echeverry-Alzate V., Martínez-Miguel E., Gómez-Senent S., Uceda S., Clemente-Suárez V.J. (2025). Bifidobacterium Infantis and Bifidobacterium Breve Improve Symptomatology and Neuronal Damage in Neurodegenerative Disease: A Systematic Review. Nutrients.

[158] Carrillo J.Á., Arcusa R., Xandri-Martínez R., Cerdá B., Zafrilla P., Marhuenda J. (2025). Impact of Polyphenol-Rich Nutraceuticals on Cognitive Function and Neuroprotective Biomarkers: A Randomized, Double-Blind, Placebo-Controlled Clinical Trial. Nutrients.

[159] Du Y., He C., An Y., Huang Y., Zhang H., Fu W., Wang M., Shan Z., Xie J., Yang Y. (2024). The Role of Short Chain Fatty Acids in Inflammation and Body Health. Int. J. Mol. Sci..

[160] Zhang S.-Y., Zhang L.-Y., Wen R., Yang N., Zhang T.-N. (2024). Histone Deacetylases and Their Inhibitors in Inflammatory Diseases. Biomed. Pharmacother..

[161] Chiang J.Y.L., Ferrell J.M. (2020). Bile Acid Receptors FXR and TGR5 Signaling in Fatty Liver Diseases and Therapy. Am. J. Physiol. Liver Physiol..

[162] Ojha S., Patil N., Jain M., Kole C., Kaushik P. (2023). Probiotics for Neurodegenerative Diseases: A Systemic Review. Microorganisms.

[163] Shah A.B., Baiseitova A., Zahoor M., Ahmad I., Ikram M., Bakhsh A., Shah M.A., Ali I., Idress M., Ullah R. (2024). Probiotic Significance of Lactobacillus Strains: A Comprehensive Review on Health Impacts, Research Gaps, and Future Prospects. Gut Microbes.

[164] Kumar A., Sivamaruthi B.S., Dey S., Kumar Y., Malviya R., Prajapati B.G., Chaiyasut C. (2024). Probiotics as Modulators of Gut-Brain Axis for Cognitive Development. Front. Pharmacol..

[165] Divyashri G., Sadanandan B., Chidambara Murthy K.N., Shetty K., Mamta K. (2021). Neuroprotective Potential of Non-Digestible Oligosaccharides: An Overview of Experimental Evidence. Front. Pharmacol..

[166] Kalyanaraman B., Cheng G., Hardy M. (2024). Gut Microbiome, Short-Chain Fatty Acids, Alpha-Synuclein, Neuroinflammation, and ROS/RNS: Relevance to Parkinson’s Disease and Therapeutic Implications. Redox Biol..

[167] Qiao L., Yang G., Wang P., Xu C. (2024). The Potential Role of Mitochondria in the Microbiota-Gut-Brain Axis: Implications for Brain Health. Pharmacol. Res..

[168] Guamán L.P., Carrera-Pacheco S.E., Zúñiga-Miranda J., Teran E., Erazo C., Barba-Ostria C. (2024). The Impact of Bioactive Molecules from Probiotics on Child Health: A Comprehensive Review. Nutrients.

[169] Yan H., Ren J., Liu G.-H. (2023). Fecal Microbiota Transplantation: A New Strategy to Delay Aging. hLife.

[170] Varesi A., Pierella E., Romeo M., Piccini G.B., Alfano C., Bjørklund G., Oppong A., Ricevuti G., Esposito C., Chirumbolo S. (2022). The Potential Role of Gut Microbiota in Alzheimer’s Disease: From Diagnosis to Treatment. Nutrients.

[171] Loh J.S., Mak W.Q., Tan L.K.S., Ng C.X., Chan H.H., Yeow S.H., Foo J.B., Ong Y.S., How C.W., Khaw K.Y. (2024). Microbiota–Gut–Brain Axis and Its Therapeutic Applications in Neurodegenerative Diseases. Signal Transduct. Target. Ther..

[172] Zhang L.-Y., Zhang S.-Y., Wen R., Zhang T.-N., Yang N. (2024). Role of Histone Deacetylases and Their Inhibitors in Neurological Diseases. Pharmacol. Res..

[173] Zhang S., Zhan L., Li X., Yang Z., Luo Y., Zhao H. (2021). Preclinical and Clinical Progress for HDAC as a Putative Target for Epigenetic Remodeling and Functionality of Immune Cells. Int. J. Biol. Sci..

[174] Xiang W., Xiang H., Wang J., Jiang Y., Pan C., Ji B., Zhang A. (2023). Fecal Microbiota Transplantation: A Novel Strategy for Treating Alzheimer’s Disease. Front. Microbiol..

[175] Novelle M.G., Naranjo-Martínez B., López-Cánovas J.L., Díaz-Ruiz A. (2025). Fecal Microbiota Transplantation, a Tool to Transfer Healthy Longevity. Ageing Res. Rev..

[176] Tian H., Wang X., Fang Z., Li L., Wu C., Bi D., Li N., Chen Q., Qin H. (2024). Fecal Microbiota Transplantation in Clinical Practice: Present Controversies and Future Prospects. hLife.

[177] Zikou E., Koliaki C., Makrilakis K. (2024). The Role of Fecal Microbiota Transplantation (FMT) in the Management of Metabolic Diseases in Humans: A Narrative Review. Biomedicines.

[178] Chance E.A., Florence D., Sardi Abdoul I. (2024). The Effectiveness of Checklists and Error Reporting Systems in Enhancing Patient Safety and Reducing Medical Errors in Hospital Settings: A Narrative Review. Int. J. Nurs. Sci..

[179] White S.L., Rawlinson W., Boan P., Sheppeard V., Wong G., Waller K., Opdam H., Kaldor J., Fink M., Verran D. (2019). Infectious Disease Transmission in Solid Organ Transplantation: Donor Evaluation, Recipient Risk, and Outcomes of Transmission. Transplant. Direct.

[180] Opara U.C., Iheanacho P.N., Li H., Petrucka P. (2024). Facilitating and Limiting Factors of Cultural Norms Influencing Use of Maternal Health Services in Primary Health Care Facilities in Kogi State, Nigeria; a Focused Ethnographic Research on Igala Women. BMC Pregnancy Childbirth.

[181] Merrick B., Allen L., Masirah M Zain N., Forbes B., Shawcross D.L., Goldenberg S.D. (2020). Regulation, Risk and Safety of Faecal Microbiota Transplant. Infect. Prev. Pract..

[182] Wang X., Zhao D., Bi D., Li L., Tian H., Yin F., Zuo T., Ianiro G., Li N., Chen Q. (2025). Fecal Microbiota Transplantation: Transitioning from Chaos and Controversial Realm to Scientific Precision Era. Sci. Bull..

[183] Kamel M., Aleya S., Alsubih M., Aleya L. (2024). Microbiome Dynamics: A Paradigm Shift in Combatting Infectious Diseases. J. Pers. Med..

[184] Nigam M., Panwar A.S., Singh R.K. (2022). Orchestrating the Fecal Microbiota Transplantation: Current Technological Advancements and Potential Biomedical Application. Front. Med. Technol..

[185] Lee B.Y., Ordovás J.M., Parks E.J., Anderson C.A., Barabási A.-L., Clinton S.K., de la Haye K., Duffy V.B., Franks P.W., Ginexi E.M. (2022). Research Gaps and Opportunities in Precision Nutrition: An NIH Workshop Report. Am. J. Clin. Nutr..

[186] Xiao Y., Feng Y., Zhao J., Chen W., Lu W. (2025). Achieving Healthy Aging through Gut Microbiota-Directed Dietary Intervention: Focusing on Microbial Biomarkers and Host Mechanisms. J. Adv. Res..

[187] Fu J., Zheng Y., Gao Y., Xu W. (2022). Dietary Fiber Intake and Gut Microbiota in Human Health. Microorganisms.

[188] Randeni N., Xu B. (2025). Critical Review of the Cross-Links Between Dietary Components, the Gut Microbiome, and Depression. Int. J. Mol. Sci..

[189] Ma Y.-Y., Li X., Yu J.-T., Wang Y.-J. (2024). Therapeutics for Neurodegenerative Diseases by Targeting the Gut Microbiome: From Bench to Bedside. Transl. Neurodegener..

[190] Liu Z., Liu M., Meng J., Wang L., Chen M. (2024). A Review of the Interaction between Diet Composition and Gut Microbiota and Its Impact on Associated Disease. J. Future Foods.

[191] Zhang J., Zhang Y., Wang J., Xia Y., Zhang J., Chen L. (2024). Recent Advances in Alzheimer’s Disease: Mechanisms, Clinical Trials and New Drug Development Strategies. Signal Transduct. Target. Ther..

[192] Sittipo P., Choi J., Lee S., Lee Y.K. (2022). The Function of Gut Microbiota in Immune-Related Neurological Disorders: A Review. J. Neuroinflammation.

[193] Mansuy-Aubert V., Ravussin Y. (2023). Short Chain Fatty Acids: The Messengers from down Below. Front. Neurosci..

[194] Toader C., Tataru C.P., Munteanu O., Serban M., Covache-Busuioc R.-A., Ciurea A.V., Enyedi M. (2024). Decoding Neurodegeneration: A Review of Molecular Mechanisms and Therapeutic Advances in Alzheimer’s, Parkinson’s, and ALS. Int. J. Mol. Sci..

[195] Mafe A.N., Büsselberg D. (2025). Modulation of the Neuro–Cancer Connection by Metabolites of Gut Microbiota. Biomolecules.

[196] Acharya A., Shetty S.S., Kumari N.S. (2024). Role of Gut Microbiota Derived Short Chain Fatty Acid Metabolites in Modulating Female Reproductive Health. Hum. Nutr. Metab..

[197] Bicknell B., Liebert A., Borody T., Herkes G., McLachlan C., Kiat H. (2023). Neurodegenerative and Neurodevelopmental Diseases and the Gut-Brain Axis: The Potential of Therapeutic Targeting of the Microbiome. Int. J. Mol. Sci..

[198] Skalny A.V., Aschner M., Gritsenko V.A., Martins A.C., Tizabi Y., Korobeinikova T.V., Paoliello M.M.B., Tinkov A.A. (2024). Modulation of Gut Microbiota with Probiotics as a Strategy to Counteract Endogenous and Exogenous Neurotoxicity.

[199] Abdelhamid M., Counts S.E., Zhou C., Hida H., Kim J.-I., Michikawa M., Jung C.-G. (2025). Protective Effects of Bifidobacterium Breve MCC1274 as a Novel Therapy for Alzheimer’s Disease. Nutrients.

[200] Sheng W., Ji G., Zhang L. (2023). Immunomodulatory Effects of Inulin and Its Intestinal Metabolites. Front. Immunol..

[201] Mahalak K.K., Firrman J., Narrowe A.B., Hu W., Jones S.M., Bittinger K., Moustafa A.M., Liu L. (2023). Fructooligosaccharides (FOS) Differentially Modifies the in Vitro Gut Microbiota in an Age-Dependent Manner. Front. Nutr..

[202] Han S.-K., Shin Y.-J., Lee D.-Y., Kim K.M., Yang S.-J., Kim D.S., Choi J.-W., Lee S., Kim D.-H. (2021). Lactobacillus Rhamnosus HDB1258 Modulates Gut Microbiota-Mediated Immune Response in Mice with or without Lipopolysaccharide-Induced Systemic Inflammation. BMC Microbiol..

[203] de Oliveira D.P., Todorov S.D., Fabi J.P. (2024). Exploring the Prebiotic Potentials of Hydrolyzed Pectins: Mechanisms of Action and Gut Microbiota Modulation. Nutrients.

[204] Mills S., Yang B., Smith G.J., Stanton C., Ross R.P. (2023). Efficacy of Bifidobacterium Longum Alone or in Multi-Strain Probiotic Formulations during Early Life and Beyond. Gut Microbes.

[205] Beteri B., Barone M., Turroni S., Brigidi P., Tzortzis G., Vulevic J., Sekulic K., Motei D.-E., Costabile A. (2024). Impact of Combined Prebiotic Galacto-Oligosaccharides and Bifidobacterium Breve-Derived Postbiotic on Gut Microbiota and HbA1c in Prediabetic Adults: A Double-Blind, Randomized, Placebo-Controlled Study. Nutrients.

[206] Nguyen N.M., Cho J., Lee C. (2023). Gut Microbiota and Alzheimer’s Disease: How to Study and Apply Their Relationship. Int. J. Mol. Sci..

[207] Binda S., Tremblay A., Iqbal U.H., Kassem O., Le Barz M., Thomas V., Bronner S., Perrot T., Ismail N., Parker J.A. (2024). Psychobiotics and the Microbiota–Gut–Brain Axis: Where Do We Go from Here?. Microorganisms.

[208] Larroya A., Pantoja J., Codoñer-Franch P., Cenit M.C. (2021). Towards Tailored Gut Microbiome-Based and Dietary Interventions for Promoting the Development and Maintenance of a Healthy Brain. Front. Pediatr..

[209] Fang P., Kazmi S.A., Jameson K.G., Hsiao E.Y. (2020). The Microbiome as a Modifier of Neurodegenerative Disease Risk. Cell Host Microbe.

[210] Zhang R., Zhang M., Wang P. (2025). The Intricate Interplay between Dietary Habits and Cognitive Function: Insights from the Gut-Brain Axis. Front. Nutr..

[211] Holmes Z.C., Villa M.M., Durand H.K., Jiang S., Dallow E.P., Petrone B.L., Silverman J.D., Lin P.-H., David L.A. (2022). Microbiota Responses to Different Prebiotics Are Conserved within Individuals and Associated with Habitual Fiber Intake. Microbiome.

[212] Chen Z., Liang N., Zhang H., Li H., Guo J., Zhang Y., Chen Y., Wang Y., Shi N. (2024). Resistant Starch and the Gut Microbiome: Exploring Beneficial Interactions and Dietary Impacts. Food Chem. X.

[213] Bianchetti G., De Maio F., Abeltino A., Serantoni C., Riente A., Santarelli G., Sanguinetti M., Delogu G., Martinoli R., Barbaresi S. (2023). Unraveling the Gut Microbiome–Diet Connection: Exploring the Impact of Digital Precision and Personalized Nutrition on Microbiota Composition and Host Physiology. Nutrients.

[214] Arafah A., Khatoon S., Rasool I., Khan A., Rather M.A., Abujabal K.A., Faqih Y.A.H., Rashid H., Rashid S.M., Bilal Ahmad S. (2023). The Future of Precision Medicine in the Cure of Alzheimer’s Disease. Biomedicines.

[215] Shukla V., Singh S., Verma S., Verma S., Rizvi A.A., Abbas M. (2024). Targeting the Microbiome to Improve Human Health with the Approach of Personalized Medicine: Latest Aspects and Current Updates. Clin. Nutr. ESPEN.

[216] Di Meco A., Vassar R. (2021). Early Detection and Personalized Medicine: Future Strategies Against Alzheimer’s Disease.

[217] Zhang T., Gao G., Kwok L.-Y., Sun Z. (2023). Gut Microbiome-Targeted Therapies for Alzheimer’s Disease. Gut Microbes.

[218] Mafe A.N., Büsselberg D. (2025). Microbiome Integrity Enhances the Efficacy and Safety of Anticancer Drug. Biomedicines.

[219] Ngah W.Z.W., Ahmad H.F., Ankasha S.J., Makpol S., Tooyama I. (2024). Dietary Strategies to Mitigate Alzheimer’s Disease: Insights into Antioxidant Vitamin Intake and Supplementation with Microbiota–Gut–Brain Axis Cross-Talk. Antioxidants.

[220] Merino del Portillo M., Clemente-Suárez V.J., Ruisoto P., Jimenez M., Ramos-Campo D.J., Beltran-Velasco A.I., Martínez-Guardado I., Rubio-Zarapuz A., Navarro-Jiménez E., Tornero-Aguilera J.F. (2024). Nutritional Modulation of the Gut–Brain Axis: A Comprehensive Review of Dietary Interventions in Depression and Anxiety Management. Metabolites.

[221] Gulliver E.L., Young R.B., Chonwerawong M., D’Adamo G.L., Thomason T., Widdop J.T., Rutten E.L., Rossetto Marcelino V., Bryant R.V., Costello S.P. (2022). Review Article: The Future of Microbiome-based Therapeutics. Aliment. Pharmacol. Ther..

[222] Wasén C., Simonsen E., Ekwudo M.N., Profant M.R., Cox L.M. (2022). The Emerging Role of the Microbiome in Alzheimer’s Disease.

[223] Pourahmad R., Saleki K., Zare Gholinejad M., Aram C., Soltani Farsani A., Banazadeh M., Tafakhori A. (2024). Exploring the Effect of Gut Microbiome on Alzheimer’s Disease. Biochem. Biophys. Rep..

[224] Ferreiro A.L., Choi J., Ryou J., Newcomer E.P., Thompson R., Bollinger R.M., Hall-Moore C., Ndao I.M., Sax L., Benzinger T.L.S. (2023). Gut Microbiome Composition May Be an Indicator of Preclinical Alzheimer’s Disease. Sci. Transl. Med..

[225] Onisiforou A., Charalambous E.G., Zanos P. (2025). Shattering the Amyloid Illusion: The Microbial Enigma of Alzheimer’s Disease Pathogenesis—From Gut Microbiota and Viruses to Brain Biofilms. Microorganisms.

[226] Liu S., Gao J., Liu K., Zhang H.-L. (2021). Microbiota-Gut-Brain Axis and Alzheimer’s Disease: Implications of the Blood-Brain Barrier as an Intervention Target. Mech. Ageing Dev..

[227] Zou Z., Lei D., Yin Y., Xu R., Luo H., Chen T., Liu M., Li X. (2025). Frequent Fecal Microbiota Transplantation Improves Cognitive Impairment and Pathological Changes in Alzheimer’s Disease FAD4T Mice via the Microbiota-Gut-Brain Axis. Heliyon.

[228] Osama A., Anwar A.M., Ezzeldin S., Ahmed E.A., Mahgoub S., Ibrahim O., Ibrahim S.A., Abdelhamid I.A., Bakry U., Diab A.A. (2025). Integrative Multi-Omics Analysis of Autism Spectrum Disorder Reveals Unique Microbial Macromolecules Interactions. J. Adv. Res..

